# Have parenting programs for disruptive child behavior become less effective?

**DOI:** 10.1111/jcpp.70049

**Published:** 2025-09-18

**Authors:** Patty Leijten, G.J. Melendez‐Torres, Sophia Backhaus, Frances Gardner, Annabeth P. Groenman, Tycho J. Dekkers, Barbara J. van den Hoofdakker, Liina Björg Laas Sigurðardóttir, Danni Liu, Marjolein Luman, Lara Mansur, Merlin Nieterau, Saskia van der Oord, Geertjan Overbeek, Constantina Psyllou, Karen Rienks, Susanne Schulz, John R. Weisz

**Affiliations:** ^1^ Research Institute of Child Development and Education University of Amsterdam Amsterdam The Netherlands; ^2^ Faculty of Health and Life Sciences University of Exeter Exeter UK; ^3^ Department of Social Policy and Intervention University of Oxford Oxford UK; ^4^ Accare Child Study Center Groningen The Netherlands; ^5^ Department of Child and Adolescent Psychiatry University Medical Center Groningen Groningen The Netherlands; ^6^ Levvel, Academic Center for Child and Adolescent Psychiatry Amsterdam The Netherlands; ^7^ Department of Clinical, Neuro and Developmental Psychology VU Amsterdam Amsterdam The Netherlands; ^8^ Faculty of Psychology and Educational Sciences KU Leuven Leuven Belgium; ^9^ Department of Psychology Harvard University Cambridge Massachusetts USA

**Keywords:** Behavioral parenting program, disruptive child behavior, systematic review, meta‐analysis, time trends

## Abstract

**Background:**

Behavioral parenting programs have been exhaustively studied over the past five decades. We used this wealth of research to examine how estimates of parenting program effects have evolved over time, and if any time trends in effect estimates can be explained by trial, sample, or intervention characteristics.

**Methods:**

We based our meta‐analysis on a systematic search of 22 international and regional databases, gray literature, and 4 trial registries for randomized controlled trials of behavioral parenting programs.

**Results:**

We identified 244 eligible trials (1,100 effect sizes; 28,916 families) from 36 countries. Parenting program effects initially reduced and then stabilized. More recent trials used more rigorous methods (e.g., more active control conditions and less risk of bias), samples that were generally older and included more girls, and evaluated interventions that on average had fewer sessions, were more often delivered by independent staff and made less use of time‐out. However, none of these developments explained the initial reduction in effect size estimates during the first decades.

**Conclusions:**

Our findings suggest that estimates of parenting program effects are currently stable: Effect sizes are no longer reducing but there is also no evidence of increases over time. Experimentation with the content, delivery, and personalization of parenting programs is needed to identify ways to increase program effects.

The first randomized controlled trials of behavioral parenting programs to reduce disruptive (i.e., oppositional, defiant, aggressive) child behavior were published in the late 1970s and early 1980s (Forgatch & Toobert, 1979; Webster‐Stratton, 1982). These studies reflected a paradigm shift in strategies to reduce disruptive child behavior—from child‐focused psychodynamic play therapy to training parents to redirect children's behavior based on operant and social learning theory principles (Patterson, 2002). In the following decades, an increasing number of randomized trials and dozens of meta‐analyses would be published (e.g., Beelmann, Arnold, & Hercher, 2023; Mingebach, Kamp‐Becker, Christiansen, & Weber, 2018). In the present study, we examine how estimates of parenting program effects have evolved over time. In addition, we examine whether any time trends can be explained by trial, sample, or intervention characteristics.

In the early 1960s, researchers at the University of Oregon broke with the tradition of treating disruptive child behavior with psychodynamic play therapy. Simple reinforcement techniques to strengthen non‐disruptive behavior yielded rapid and strong improvements in children's behavior (Patterson, 1965), especially when combined with non‐violent negative consequences for disruptive behavior, such as time‐out and taking away privileges (Wells, 1997). This led to programs that trained parents to change their behavior in order to reduce disruptive child behaviors. In the following years, the Oregon team and investigators from other parts of the United States built theories and programs on how parents can alter the development of disruptive child behavior. A notable example was Patterson's model of coercive interaction cycles, explaining how parents and children can unwittingly reinforce aversive behavior in each other, leading to interaction cycles that become increasingly difficult to break (Patterson, 1982). This model, which has been widely supported empirically (e.g., Lunkenheimer, Lichtwarck‐Aschoff, Hollenstein, Kemp, & Granic, 2016; Smith et al., 2014), combined with Hanf's model stressing the need to strengthen parent–child relationship quality in families with disruptive child behavior (Hanf, 1969), forms the basis of most established parenting programs to prevent or treat disruptive child behavior (Kaehler, Jacobs, & Jones, 2016; Reitman & McMahon, 2013; Weisz & Kazdin, 2017).

Now, more than half a century later, behavioral parenting programs are implemented across the globe (Backhaus, Leijten, Jochim, Melendez‐Torres, & Gardner, 2023) and every year new meta‐analyses synthesize the findings of an increasing number of trials (e.g., Beelmann et al., 2023). This wealth of research places us in the unique position to examine how estimates of parenting program effects have evolved. Changes in estimates of parenting program effects can reflect changes in research methods (e.g., worse or better precision in estimating the true effects) but also changes in the true effects of parenting programs. Given the prevalence and negative consequences of disruptive behavior problems in childhood (Fairchild et al., 2019), it is important to know whether the effects of parenting programs—the recommended strategy to prevent and treat these problems—have improved, declined, or remained relatively stable across the years as research has accumulated.

Evidence from trials on interventions for child and adolescent conduct problems suggests that intervention effects have gradually reduced over five decades (Weisz et al., 2019). This trend is not unique to interventions for conduct problems. A similar trend, with mainly initial reductions in effect sizes, has been observed in cognitive behavioral therapy for adult depression (Ljótsson, Hedman, Mattsson, & Andersson, 2017). However, trials on interventions for anxiety show a different trend: Effect sizes of cognitive behavioral therapy for adult anxiety have not changed over time (Hofmann, Kasch, & Reis, 2025), but effect sizes for psychotherapy for child and adolescent anxiety have increased over time (Weisz et al., 2019).

The reasons for changes in intervention effects are not well understood. Suggested explanations for reduced effect sizes over time include that trials have become more rigorous over time, with larger samples, less risk of bias, and more active control groups. Several meta‐analyses indeed show that less rigorous trials indeed yield larger effect sizes (e.g., Cuijpers, van Straten, Bohlmeijer, Hollon, & Andersson, 2010; Gellatly et al., 2007). In addition, small samples increase the likelihood of chance findings, and of these, the larger positive effects may have been more likely to get published. It is also well known that active control groups tend to yield smaller between‐group effects (Rifkin, 2007). Researcher allegiance may have increased effect sizes especially in some of the earlier trials (Leykin & DeRubeis, 2009; Ljótsson et al., 2017). Researcher allegiance effects can be caused by biases—distortion of findings because of program developers' preferences (Luborsky et al., 1999), but also by better expertise and skill with the target treatment, and by accumulation of evidence from prior studies indicating that a particular treatment is in fact more effective (Hollon, 1999).

Alternative explanations include that the structure of interventions, often weekly in‐person sessions, may fit less well with societal life today than it did several decades ago (Weisz et al., 2019). The use of smartphones may have made individuals accustomed to having help at hand more immediately. Although an increasing number of interventions use technology to support flexible delivery (e.g., Jones et al., 2021), many still use the traditional structure. In addition, most parents nowadays have better access to online information about children's development and parenting (Canário et al., 2022), making the content of parenting programs less novel and therefore perhaps less likely to have a big impact. Also, attempts to increase program effects may have led scholars to add new elements to programs, while evidence suggests that stacking elements is often counter effective (Bakermans‐Kranenburg, Van Ijzendoorn, & Juffer, 2003; Leijten, Melendez‐Torres, & Gardner, 2022). Last, it may be that the factors contributing to youth mental health problems (e.g., social media influences) are changing faster than our treatments are able to change to address those factors (Weisz et al., 2019).

For parenting programs, time trends in effect sizes are unknown. There may be similar declines as in the broader field of interventions for youth conduct problems (Weisz et al., 2019). In addition, some effective techniques to increase children's compliance, most notably time‐out, have been under scrutiny (Readdick & Chapman, 2000; Siegel & Bryson, 2014) and may be used less in more recently evaluated programs. Also, given the global dissemination of parenting programs in the past decades (Backhaus et al., 2023), there may be changes in sample characteristics that influence program effect sizes (e.g., baseline problem severity or cultural fit; Leijten et al., 2020; Morelli et al., 2018).

However, there may also be reasons to be hopeful that parenting programs have become more effective in recent years. The field has invested in improving our understanding of the program elements that are most likely to contribute to effective change (e.g., Kaminski, Valle, Filene, & Boyle, 2008; Leijten et al., 2019), in adapting transported programs to local contexts while maintaining functional fidelity (Baumann et al., 2015), and in technological developments allowing for more versatile delivery formats that may better fit families' needs (e.g., Duppong‐Hurley, Hoffman, Barnes, & Oats, 2016). The goal of these investments was to provide more effective programs.

## The present study

We examined how estimates of the effects of parenting programs on disruptive child behavior have evolved over time. Specifically, we used meta‐analysis to identify associations between the year in which the trial was conducted, effect size, and trial, sample, and intervention characteristics to examine (1) linear and non‐linear time trends in estimated parenting program effects on reduced disruptive child behavior; (2) how trial, sample, and intervention characteristics relate to time and effect size; and (3) whether any time trends in estimated effect sizes could be accounted for by time trends in trial, sample, and intervention characteristics. Our analyses were exploratory, guided by research questions rather than hypotheses.

## Methods

### Information sources and search strategy

Our primary source of studies was the preregistered systematic literature review by Backhaus et al. (2023; preregistered on PROSPERO: CRD42019141844; we did not pre‐register separately), based on an extensive search by five researchers in 22 databases (e.g., PsycINFO and MEDLINE), including various non‐English databases (e.g., Biomedical journals from India and Russia), gray literature (e.g., ProQuest, China Doctoral Dissertations Full‐Text Database), and trial registries (e.g., ClinicalTrials.gov and WHO International Clinical Trials Registry Platform), last updated in May 2024. The full search string is in Appendix S1. We also searched the reference lists of the included studies for any additional eligible studies.

### Eligibility criteria and selection process

We included (i) randomized controlled trials that compared the effects of (ii) a behavioral parenting program on (iii) disruptive child behavior against (iv) any control. We focused on trials including samples of (v) children with a mean age between 2 and 10 years because conduct problems in adolescence require a more systemic approach (Boldrini, Ghiandoni, Mancinelli, Salcuni, & Solmi, 2023). Randomized crossover studies were also excluded.

We defined behavioral parenting programs as the program content being primarily based on operant and social learning principles. These programs typically teach parents to use differential attention and reinforcement (e.g., reinforcing positive behavior and avoiding reinforcement of disruptive behavior). They vary in the extent to which they complement this approach with other intervention components (e.g., increasing parent–child relationship quality, emotion regulation, and parental self‐management; Kaehler et al., 2016). We excluded trials that combined a parenting program with a child or teacher program within one trial arm.

Disruptive child behavior had to be an intervention target for the study to be included in our meta‐analysis, but it did not have to be the primary target. We defined disruptive behavior as oppositional and defiant behavior and conduct problems (e.g., aggression). We therefore included measures such as the Eyberg Child Behavior Inventory (ECBI; Eyberg & Ross, 1978), the Conduct Problems subscale of the Strengths and Difficulties Questionnaire (SDQ; Goodman, 1997), and the Aggressive Behaviors subscale of the Child Behavior Checklist (CBCL; Achenbach, 1991). Some of these measures include items on ADHD in addition to items on oppositional and defiant behavior and conduct problems (e.g., ECBI), but we excluded measures that exclusively focused on ADHD (e.g., the Hyperactivity/inattention subscale of the SDQ).

Because of the strong overlap among oppositional and defiant behavior, conduct problems, and attention‐deficit/hyperactivity disorder (ADHD) symptoms in young children (Hinshaw, Lahey, & Hart, 1993), as well as similarities in the programs used to treat these problems, we included trials that specifically targeted children with ADHD. However, to ensure sufficient homogeneity, we excluded trials that specifically targeted children with autism spectrum disorder, intellectual disabilities, or medical conditions (e.g., traumatic brain injury). Parenting programs to treat behavioral problems in these children are often condition‐specific.

### Data items and collection process

Three team members independently extracted data items using a piloted data extraction form with coder disagreements resolved through discussion. In addition to the study variables (described in detail below), we coded trial characteristics for descriptive purposes (e.g., country and its World Bank income status). In a random sample of 27% of the trials from the most recent update of our systematic search, inter‐rater agreement was 98%.

### Time

We coded for each trial the median year in which the data for the trial were collected (e.g., if data were collected between 2009 and 2011, we coded trial year as 2010). For studies where the year of data collection was missing, we used the average time lag between the year of publication and the year of data collection (3.77 years, rounded to 4 years) to estimate the year in which the trial was conducted (e.g., if year of data collection was missing for a trial published in 2013, we coded trial year as 2009). The year of data collection ranged from 1975 to 2022. We divided the year by 10, to facilitate the interpretation of regression coefficients. Last, we grand mean centered the year and computed a squared year, to explore the possibility of non‐linear trends.

### Effect size

We computed Cohen's *d* based on post‐intervention means and standard deviations, subtracting children's mean level of disruptive behavior in the control condition from children's mean level of disruptive behavior in the intervention condition, and dividing this difference by the pooled post‐intervention standard deviation. Where means and standard deviations were not reported, we used relevant model statistics such as regression coefficients and *F*‐values and converted these to Cohen's *d* using the Campbell Collaboration calculator (https://www.campbellcollaboration.org/calculator/). To reduce the effect of possibly spurious outliers, outliers were winsorized and replaced with values three standard deviations from the mean effect size. We did not conduct baseline adjustment because many trials (and especially older trials) did not report data for this.

### Trial characteristics

We coded (i) sample size (i.e., total number of participants at baseline), (ii) type of control condition (0 = *no intervention or waitlist, labeled ‘passive control’*; 1 = *low intensity intervention or unstandardized treatment as usual, labeled ‘active control’*), (iii) developer involvement (0 = *no developer of the intervention co‐authored the publication of the trial findings*; 1 = *a developer of the intervention co‐authored the publication*), (iv) preregistration in a trial register (e.g., clinicaltrials.gov) or preregistered analysis plan on a preregistration platform (e.g., Open Science Framework or AsPredicted) (0 = *no*; 1 = *yes*), (v) the use of an observational (versus only parent‐reported) outcome of disruptive child behavior (0 = *no*; 1 = *yes*), and (vi) risk of bias using the Cochrane Risk of Bias Tool 1.0 for randomized controlled trials (Higgins et al., 2011): random sequence generation, random sequence concealment, blinding of participants, missing outcome data, and selective outcome reporting. For each indicator, we rated risk of bias as 0 (*low*), 1 (*unclear*), or 2 (*high*). We used version 1.0 of the Risk of Bias Tool, rather than version 2.0, because version 1.0 focuses on trial‐level rigor and version 2.0 focuses on outcome‐level rigor; the focus of our study is on trial characteristics.

### Sample characteristics

We coded (i) children's mean age in years at baseline, (ii) child sex or gender (i.e., percentage of boys in the sample), (iii) baseline severity of disruptive child behavior (i.e., mean Eyberg Child Behavior Inventory (ECBI)—Intensity Scale score in the intervention condition; 48% of the trials used the ECBI, all other measures were used less frequently), (iv) socioeconomic privilege (0 = *sample primarily disadvantaged*; 1 = *sample primarily non‐disadvantaged*), based on available information in the trial reports on family income, education, and employment; and (v) the sample was culturally and/or ethnically different from the sample for which the intervention was originally developed (0 = *no*; 1 = *yes*).

### Intervention characteristics

We coded (i) number of sessions, (ii) delivery staff (0 = *research team*; 1 = *independent staff*), (iii) group versus individual delivery (0 = *group*; 1 = *individual*), (iv) self‐directed versus therapist‐led delivery (0 = *self‐directed*; 1 = *therapist‐led*), (v) use of in vivo coaching or video feedback (0 = *no*; 1 = *yes*), and (vi) whether the intervention included the following elements (each coded as 0 = *no*; 1 = *yes*): reinforcement of positive child behavior, avoiding reinforcement of disruptive child behavior (including separate codes for time‐out, ignore, and the use of logical/natural consequences or removing privileges), relationship enhancement techniques (including a separate code for child‐led play), parental self‐management (e.g., relaxation or increasing social support), and teaching parents how to enhance children's broader skills (e.g., teaching children social or problem‐solving skills).

### Data synthesis

We used robust variance estimation (Hedges, Tipton, & Johnson, 2010) in Stata version 18.0 (robumeta package) to estimate (1) an intercept only model with the overall program effect sizes; (2) a series of single predictor models with trial year predicting effect size, as well as each of the individual trial, sample, and intervention characteristics predicting trial year and effect size; and (3) a series of multi‐predictor models, with year and trial, sample, and intervention characteristics predicting effect sizes. We used robust variance estimation also for descriptive purposes, including group differences in effect sizes. For example, we estimated effect size differences between middle‐income and high‐income countries by predicting effect size with a dummy variable indicating whether the effect size came from a trial conducted in a middle‐income country (score of 0) versus in a high‐income country (score of 1). Robust variance estimation weighs the multiple effect sizes included in each trial using an approximate variance–covariance matrix (Hedges et al., 2010). Within‐trial correlation of effect sizes was set at the default of *ρ* = .80. Data and code are available on Open Science Framework: https://osf.io/qbjgp/.

## Results

### Included trials

**Figure 1:**
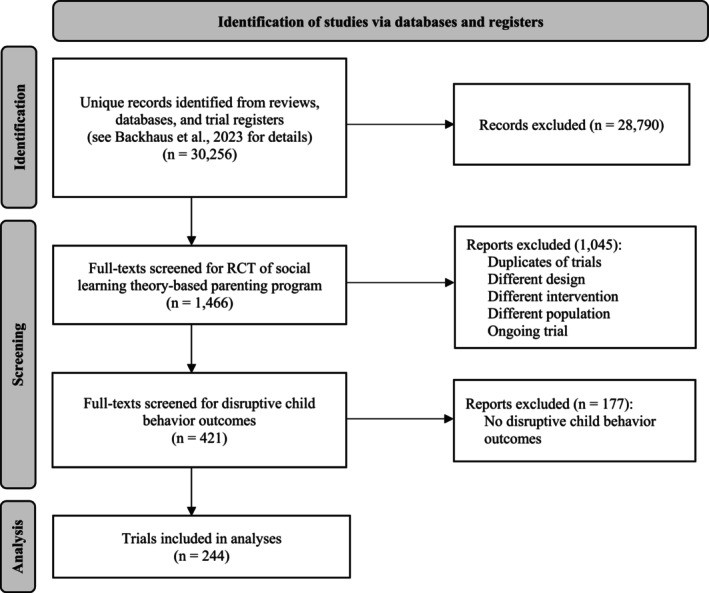
Flowchart of data collection procedure

Our systematic search yielded 30,256 unique hits (Figure 1). Four researchers screened all titles and abstracts. The same researchers then examined 1,466 full‐text papers, dissertations, and trial registrations, of which 244 trials with 1,100 effect sizes based on 28,916 families were eligible for inclusion (see Appendix S2 for included trials and their characteristic; the PRISMA checklist is in Appendix S3). Of these 244 trials, 19% were included by Weisz et al. (2019), indicating the different scope of the two reviews (e.g., our inclusion of prevention trials, searching 26 international databases and including non‐English language reports).

The overall effect of parenting programs on disruptive child behavior was *d* = −0.36 (95% CI [−0.42, −0.31]). Converting this to a common language effect size (i.e., the probability for families to be better off in the intervention group than in the control group; Magnusson, 2022) suggests that any family participating in a parenting program had a likelihood of 60% to experience significantly fewer disruptive behaviors than any family not participating in a parenting program.

Trials were conducted in 36 different countries from all six World Bank regions. Initial overrepresentation by the United States (until 2000, 74% of trials was conducted in the United States) gradually shifted to representation by more countries. Since 2015, around one third of the trials were conducted in lower and upper middle‐income countries. This is an encouraging increase from 2% before 2000 and 9% between 2000 and 2015, but still a severe underrepresentation of the 75% of the world population living in middle‐income countries (World Bank, 2024). Low‐income countries (8.5% of the world population; World Bank, 2024) are also seriously underrepresented with only one trial in Liberia (Sim et al., 2014). Effect sizes in high‐income countries did not differ from those in lower and upper middle‐income countries (differential *d* = 0.11 (95% CI [−0.14, 0.36])).

### Time trends in effect sizes

**Figure 2:**
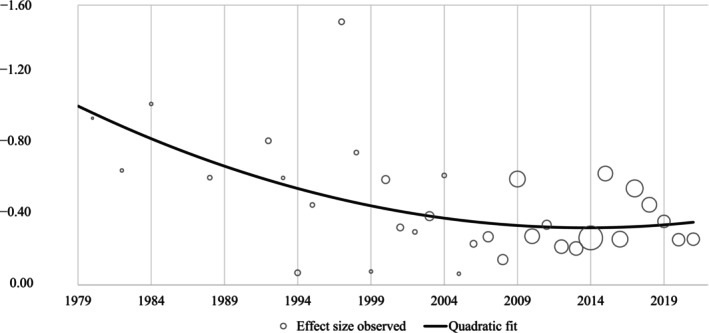
Time trend in parenting program effects (Cohen's *d*) on disruptive child behavior (bubble size reflects number of trials)

Estimates of parenting program effects on disruptive child behavior have become smaller over time, in a non‐linear fashion (linear coefficient = 0.02, 95% CI [−0.05, 0.10]; quadratic coefficient = −0.05, 95% CI [−0.10, −0.01]): Effect sizes reduced during the first decades and then stabilized around the year 2000 (Figure 2). There was no clear evidence of increases in program effects.

### Time trends in trial, sample, and intervention characteristics

More recent trials used more active control conditions, were less often conducted by the program developer, more often preregistered, less likely to include observed measures of disruptive child behavior, and had less risk of bias (Table 1). There were no significant changes over time in sample size. Of the trial characteristics that changed over time, only the use of an active control condition, preregistration, and risk of bias in terms of random allocation concealment were associated with program effect size. Effect sizes were smaller in trials with an active control condition, in trials that were preregistered, and in trials with less risk of bias regarding random allocation concealment (Table 1).

**Table 1:** Associations of trial, sample, and intervention characteristics with time and effect size

	Regression coefficient for association with time	Regression coefficient for association with effect size^a^
Trial characteristics (*n =* trials included in analysis)		
Sample size (*n* = 244) N *at baseline*	0.02 [−0.001, 0.03]	
Active control condition (*n* = 243) *0 = no; 1 = yes*	**4.25 [1.50, 7.00]**	**0.17 [0.07, 0.27]**
Developer involvement (*n* = 243) *0 = no; 1 = yes*	**−4.06 [−6.27, −1.85]**	−0.002 [−0.12, 0.11]
Preregistration (*n* = 244) *0 = no; 1 = yes*	**10.33 [8.58, 12.08]**	**0.12 [0.01, 0.23]**
Observational outcome (*n* = 244) *0 = no; 1 = yes*	**11.46 [−16.26, −6.66]**	0.03 [−0.19, 0.26]
Risk of bias (*n* = 244) *0 = low; 1 = unclear; 2 = high*		
Random sequence allocation	**−6.23 [−8.79, −3.66]**	−0.04 [−0.14, 0.07]
Random sequence concealment	**−3.40 [−5.51, −1.30]**	**−0.11 [−0.19, −0.02]**
Blinding of participants	Insufficient variation	
Incomplete data	**−2.82 [−5.01, −0.64]**	−0.03 [−0.10, 0.05]
Selective outcome reporting	**−5.63 [−7.76, −3.50]**	−0.08 [−0.16, 0.01]
Sample characteristics		
Child age (*n* = 243) *Mean sample age in years*	**0.84 [0.08, 1.34]**	0.005 [−0.02, 0.03]
Child sex (*n* = 224) *Proportion of boys*	**−21.11 [−31.21, −11.02]**	−0.48 [−0.96, 0.01]
Child disruptive behavior severity (*n =* 124) *Baseline ECBI in intervention condition*	**−0.05 [−0.09, −0.001]**	−0.004 [−0.01, 0.00]
Family socioeconomic status (*n* = 215) *0 = primarily disadvantaged; 1 = not primarily disadvantaged*	1.64 (1.35) [−1.03, 4.31]	
Cross‐cultural transportation (*n* = 244) *0 = similar population; 1 = different country/culture/ethnic group*	**4.53 (1.13) [2.29, 6.76]**	0.02 (0.06) [−0.08, 0.13]
Intervention characteristics		
Number of sessions (*n* = 239) *Number of sessions offered*	**−0.22 (0.08) [−0.40, −0.05]**	0.002 (0.01) [−0.01, 0.01]
Delivery staff (*n* = 206) *0 = research team; 1 = independent staff*	**4.97 (1.23) [2.53, 7.40]**	**0.11 (0.06) [0.005, 0.23]**
Delivery mode (*n* = 243)		
Individual (vs *group*)	−1.29 (1.60) [−4.48, 1.89]	
Therapist‐led (vs *self‐directed*)	−2.61 (1.74) [−6.10, 0.89]	
In vivo coaching or video feedback (*0 = no; 1 = yes*)	−0.55 (2.19) [−5.36, 4.26]	
Components (*n* = 241) (*0 = no; 1 = yes*)		
Positive reinforcement	Insufficient variation	
Avoiding reinforcement	−6.42 (3.17) [−13.88, 1.04]	
Time‐out	**−6.03 (1.70) [−9.47, −2.59]**	−0.05 (0.07) [−0.20, 0.09]
Ignore	−2.70 (1.55) [−5.79, 0.38]	
Consequences	0.31 (2.05) [−3.81, 4.43]	
Relationship building	2.32 (1.65) [−0.96, 5.60]	
Child‐led play	−2.48 (1.32) [−5.08 0.13]	
Parental self‐management	−1.47 (1.32) [−4.07, 1.14]	
Parents teaching children skills	−0.69 (1.26) [−3.16, 1.79]	

Coefficients in bold are significant at α = .05.

^a^ Associations with effect size were tested only for variables associated with time.

More recent trials included fewer boys and older children, and more often families that were culturally or ethnically different from families for which the intervention was first studied. There was no time trend in family socioeconomic disadvantage (Table 1). None of the sample characteristics that changed over time were associated with program effect sizes.

More recent trials evaluated programs that on average consisted of fewer sessions, were more often delivered by independent staff (rather than staff from the investigator team), and less often taught time‐out. None of the other intervention characteristics changed over time (Table 1). The number of sessions and the use of time‐out were not associated with effect size, but programs delivered by independent staff yielded on average smaller effects than programs delivered by staff from the investigator team.

### Do time trends in trial, sample, and intervention characteristics account for time trends in effect sizes?

We tested whether the trial, sample, and intervention characteristics that were associated with both time and effect sizes (i.e., active control, preregistration, risk of bias regarding allocation concealment, and delivery staff) accounted for the time trend in effect sizes. Correlations between these four variables were weak to moderate (ranging .10 to .30), suggesting relative independence. We tested these four in both univariate and multivariate meta‐regressions. In all cases, the quadratic time trend remained significant (Table 2), suggesting that reductions in effect sizes during the first decades were not driven by the included trial, sample, and intervention characteristics.

**Table 2:** Trial and sample characteristics explaining time trends in effect sizes

	Regression coefficients
Individual predictor models (*n =* trials included in analysis)	
Year (linear)	0.01 (0.04) [−0.07, 0.08]
Year (quadratic)	**−0.06 (0.02) [−0.10, −0.01]**
Active control (*n* = 243)	**0.17 [0.07, 0.27]**
Year (linear)	−0.02 (0.04) [−0.11, 0.07]
Year (quadratic)	**−0.07 (0.02) [−0.12, −0.02]**
Preregistration (*n* = 244)	0.14 [−0.0001, 0.27]
Year (linear)	0.01 (0.03) [−0.06, 0.08]
Year (quadratic)	**−0.05 (0.02) [−0.10, −0.01]**
Random sequence concealment (*n* = 244)	**−0.10 (0.04) [−0.18, −0.01]**
Year (linear)	−0.02 (0.04) [−0.10, 0.06]
Year (quadratic)	**−0.07 (0.03) [−0.12, −0.02]**
Delivery staff (*n* = 206)	0.09 (0.06) [−0.03, 0.21]
Combined predictor model (*n =* 205)	
Year (linear)	−0.07 [−0.18, 0.03]
Year (quadratic)	**−0.09 [−0.15, −0.03]**
Active control	0.12 [−0.002, 0.25]
Preregistration	0.10 [−0.06, 0.26]
Random sequence concealment	−0.06 [−0.15, 0.04]
Delivery staff	0.07 [−0.05, 0.19]

Coefficients in bold are significant at α = .05.

## Discussion

We examined time trends in the estimated effect sizes of behavioral parenting programs for disruptive child behavior. Effect sizes declined during the earlier years (1970s and 1990s) and then stabilized (2000s–2020s). Declines in effect sizes could not be explained by time trends in trial characteristics (e.g., more active control conditions and preregistration over time), sample characteristics (e.g., older children and more girls over time), or intervention characteristics (e.g., fewer sessions and less use of time‐out over time). There was no evidence of increases in program effects in later years.

Our finding that estimates of effect sizes were reduced over time matches earlier findings on the effects of different types of psychotherapy for child and adolescent conduct problems (Weisz et al., 2019). In addition, the identified non‐linear trend matches the trend observed for psychotherapy for adult depression (Ljótsson et al., 2017). Similar to Weisz et al. (2019), we were unable to identify changes in trial, sample, and intervention design that accounted for this time trend, despite testing a range of indicators of trial rigor that we know have improved over time and that are associated with smaller estimates of intervention effects (e.g., more active control conditions and less risk of bias; Cuijpers, Harrer, Miguel, Ciharova, & Karyotaki, 2025; Weisz et al., 2017).

The observed initial reduction in effect sizes might possibly be explained by changes in trial, sample, and intervention characteristics that we did not include in our analyses. For example, trials provided little information on the percentage of children in the sample with comorbid mental health problems and on the time and training and competencies of the staff delivering the intervention. Alternatively, the reduction in effect sizes may reflect an actual reduction in how much families benefit from parenting programs. Much has changed in society, family life and parenting, and information transmission in the past decades. It might well be that a variety of societal, cultural, and technical changes in the advent of the current millennium have contributed to the relatively stable reduction in the effects of behavioral parent training programs. For example, how parents perceive and implement support from experts may be influenced by parents' access to online information about disruptive child behavior and parenting (Dworkin, Connell, & Doty, 2013), by trends towards intensive and perfectionistic parenting in some countries (Faircloth, 2023), and by parental feelings of confidence or burn‐out in their parenting role, which—seemingly paradoxically—are both higher in current than in previous generations (Mikolajczak, Gross, & Roskam, 2019; Pew Research Center, 2015). Future investigations of time trends in the effects of parenting programs ideally incorporate data on societal characteristics to complement our analyses of trial characteristics.

There was no evidence that program effects are on the rise in recent years. Insights into, for example, the most effective ingredients of parenting programs (e.g., Leijten et al., 2019) and the use of technology to support parents' participation (Jones et al., 2021) are still relatively recent and may need more time to start influencing program effects. Also, it might be that as long as programs rely on the same core principles, effect sizes will remain largely the same (Jones, Mair, Kuppens, & Weisz, 2019; Weisz et al., 2019). That said, alternative parenting program approaches to reducing disruptive child behavior have been evaluated (e.g., emotion coaching, Havighurst et al., 2013; attachment enhancement, Stattin, Enebrink, Özdemir, & Giannotta, 2015; mindfulness, Coatsworth et al., 2015), but none of these outperform conventional behavioral parenting programs in terms of their effects on disruptive child behavior.

Strengths of our study include that the data span more than four decades, reflect studies in 36 different countries from all six World Bank regions, were not limited to English language reports, and included 1,100 effect sizes from 244 trials of programs based on the same underlying theoretical principles. This yielded meaningful variation in key factors (i.e., time and trial characteristics), while keeping other factors relatively constant (e.g., the main content of the intervention). In addition, our examination of both linear and non‐linear associations revealed that effect sizes declined only in the earlier decades. Last, we considered potentially confounding factors at the level of trial design, sample characteristics, and intervention content and delivery that have been shown to predict intervention effect sizes (Leijten et al., 2019; Luborsky et al., 1999).

Limitations of our study include that our findings reflect associations between developments over time; we cannot rule out the possibility that other, or additional, historical developments caused effect sizes to decrease. In addition, we were limited by the trial, sample, and intervention characteristics reported in the original studies. Especially for sample characteristics, aggregate‐level meta‐analysis like the one presented here masks meaningful variation within samples because it relies on trial‐level data (e.g., percentage of boys and children's mean age) rather than the characteristics of individual children. For some predictors, confidence intervals were relatively wide (e.g., for active control and observational measures), suggesting that some of our predictor analyses had limited statistical power. Trial characteristics may also interact in ways that we were not able to discern with the available data. We also did not conduct baseline adjustment because many trials did not report data for this. This may have led to less precise estimates of effect sizes, especially in smaller trials, where baseline inequivalence is more likely to occur. Last, because the number of trials published in the first decades is much smaller than the number of trials published in the last few decades (Figure 2), estimates of program effects in the earliest years may be less reliable.

From a clinical perspective, it is reassuring for families seeking help for disruptive child behavior that parenting programs seem to have maintained their level of effectiveness in recent decades, despite increased scientific rigor and societal changes. However, overall effect sizes are small: Families had an overall likelihood of 60% to be better off with than without a parenting program. In addition, overall effect sizes provide little insight into how much individual families benefit from parenting programs. Heterogeneity in intervention benefit is large and overall effects mask that some families do not benefit at all (i.e., these families may still improve, but not more than they would in the control condition) while others benefit greatly (e.g., Van Aar et al., 2019). Despite intensive efforts, parenting program research still struggles to accurately predict which families will benefit. The only consistent predictor is the severity of children's disruptive behavior at baseline—families with more severe problems tend to benefit more (McMahon, Goulter, & Frick, 2021), potentially because more severe problems at baseline mean there is more room for improvement, although there is some evidence for an upper limit to this trend (Laas Sigurðardóttir et al., 2024).

From a scientific perspective, our findings warrant reservation about conducting more trials of the same parenting programs in similar settings—they may only confirm what that hundreds of randomized trials and dozens of meta‐analyses have already established. If we aspire to increase the effects of parenting programs, experimentation with program content, delivery, and personalization, based on insights from basic mental health science (Holmes, Craske, & Graybiel, 2014; Ng & Weisz, 2016), is needed. In terms of program content, our understanding of the key ingredients of behavioral parenting programs could lead to leaner versions of programs that are more versatile for experimenting with the change, addition, or removal of elements (Leijten, Weisz, & Gardner, 2021). In addition, experimenting can be done with the delivery of parenting programs, such as using a more flexible structure (e.g., yearly checkups; Dishion et al., 2008), and with assigning program content based on individual family characteristics. Such scientific exercises can only be done if program manuals are made available via open access, which is increasingly common, but still not the norm (Cuijpers, Boyce, & van Ommeren, 2024).

Furthermore, rigorous experimentation with intervention content, delivery (e.g., therapist competencies), and personalization requires a diverse set of evaluation designs. Randomized trials with pre‐ and post‐intervention assessments are powerful for estimating overall program effects but less useful for identifying subtle but meaningful impacts of changes to program content or delivery (Leijten et al., 2021). Technology has made frequent assessments easier, allowing for research designs that track families' progress during the intervention to better understand when changes occur. Tracking families' progress during interventions for youth mental health is not new (Chorpita et al., 2008; Deković, Asscher, Manders, Prins, & van der Laan, 2012), but underused. Another promising development is integrative data analysis (i.e., pooling data from different trials), which can yield new insights into different mechanisms underlying change in different families (Laas Sigurðardóttir et al., 2025). These insights can be used to better tailor and target programs. The next few decades will show whether these innovations translate into improvements in the effects of parenting programs on disruptive child behavior.

## Ethical considerations

We meta‐analyzed the results of previous trials. We did not have access to any participant‐level data. Ethical approval was therefore not applicable.

**Key points**

**What's known?**

- It has been 50 years since the first randomized evaluations of behavioral parenting programs.

**What's new?**

- Estimates of parenting program effects on disruptive behavior initially reduced and have remained stable since the new millennium.
- Reductions in effect sizes could not be fully explained by increased trial rigor, sample, or parenting program characteristics.

**What's relevant?**

- Experimentation with the content, delivery, and personalization of parenting programs is needed to identify ways to increase program effects.

## Supporting information


**Appendix S1.** Search string have parenting programs for disruptive child behavior become less effective? (Leijten et al., 2025; *Journal of Child Psychology and Psychiatry*).

**Appendix S2.** Included trials. Have parenting programs for disruptive child behavior become less effective? (Leijten et al., 2025; *Journal of Child Psychology and Psychiatry*).

**Appendix S3.** PRISMA checklist. Have parenting programs for disruptive child behavior become less effective? (Leijten et al., 2025; *Journal of Child Psychology and Psychiatry*).

## Data Availability

The data that support the findings of this study are openly available on the Open Science Framework (https://osf.io/qbjgp).
