# Supplementary material for: Have parenting programs for disruptive child behavior become less effective?
Source: J Child Psychol Psychiatry. 2025 Sep 18;67(1):127–37. doi: 10.1111/jcpp.70049 (PMC12699127; doi:10.1111/jcpp.70049)
Supplement: Supplementary file 1 — Appendix S1. Search string have parenting programs for disruptive child behavior become less effective? (Leijten et al., 2025; Journal of Child Psychology and Psychiatry). [file JCPP-67-127-s001.docx]

**Appendix I: Search String**

Have Parenting Programs for Disruptive Child Behavior Become Less Effective?

(Leijten et al., 2025; *Journal of Child Psychology and Psychiatry*)

The present meta-analysis built on the systematic review by Backhaus et al. (2023) that searched for trials of the effects of parenting programs based on social learning theory principles. The following excerpt from their Supplemental Materials provides an example of the full search strings used in their study.

For the purposes of the present meta-analysis, we updated the systematic review by Backhaus et al. (2023) in May 2024.

We took the following essential concepts of the inclusion criteria to develop the search string. These terms were combined using Boolean logic (“or”, “and”). Four conceptual categories with relevant terms were used: 1) intervention, 2) parenting (including abuse), 3) child behavioural and emotional problems, 4) we did not use a term for RCT, as there is evidence that too many RCTs are missed by employing terms for the study design (Higgins & Green, 2008)

As an example, the search terms for MEDLINE:

1. ((parent$ or famil$) adj (program$ or intervention$ or training or education or group)).tw.

2. behavior therapy/ or cognitive therapy/

3. (behavio#r adj3 (train$ or intervention$ or therap$ or program$)).tw.

4. (cbt or cognitive behavio#ral therapy).tw.

5. (cognitive adj3 (therap$ or intervention$ or train$ or program$)).tw.

6. (triple p or positive parenting program).ti,ab,kw.

7. incredible years.ti,ab,kw.

8. PCIT.mp. or (Parent-child adj interaction adj therap$).ti,ab,kw. [mp=title, abstract, original title, name of substance word, subject heading word, floating sub-heading word, keyword heading word, organism supplementary concept word, protocol supplementary concept word, rare disease supplementary concept word, unique identifier, synonyms]

9. PMT.mp. or (parent adj management adj training).ti,ab,kw. [mp=title, abstract, original title, name of substance word, subject heading word, floating sub-heading word, keyword heading word, organism supplementary concept word, protocol supplementary concept word, rare disease supplementary concept word, unique identifier, synonyms]

10. (family adj check-up).ti,ab,kw.

11. 1 or 2 or 3 or 4 or 5 or 6 or 7 or 8 or 9 or 10

12. conduct disorder$.mp.

13. (oppositional adj3 (defiant$ or disorder$)).mp.

14. (conduct adj3 (difficult$ or disorder$ or problem$)).mp.

15. (behavio#ral adj3 (problem$ or difficult$ or disorder$)).mp.

16. aggressive behavio#r$.mp.

17. (emotional adj1 behavio#ral problem$).mp.

18. (child$ adj3 behavio#r$ disorder$).mp.

19. social behavio#r disorder$.mp.

20. ((antisocial or externali$ or internali$ or disruptive) adj (behavio#r or problem$ or difficult$)).mp. [mp=title, abstract, original title, name of substance word, subject heading word, floating sub-heading word, keyword heading word, organism supplementary concept word, protocol supplementary concept word, rare disease supplementary concept word, unique identifier, synonyms]

21. ((child adj abus$) or maltreat$ or (psychological adj aggression) or neglect or (corporal adj punish$)).mp.

22. ((exp parenting skills/ or exp disciplin$/ or exp emotio$/) adj regulation/) or exp warmth/ or parenting/ or exp Mother Child Communica$/ or exp Child Disciplin$/ or exp Father Child Relation$/ or exp Mother Child Relation$/ or exp Parent Child Relation$/ or exp Parent Child Communicati$/ or exp Father Child Communicat$/ or exp child parent relation$/ or exp child rearing/ or exp family functioning/ or exp family conflict/ or exp maternal behavio#r/ or exp paternal behavio#r/

23. 12 or 13 or 14 or 15 or 16 or 17 or 18 or 19 or 20 or 21 or 22

24. 11 and 23

25. limit 24 to yr="2014 -Current"

**Full reference to the systematic review by Backhaus et al. (2023):**

Backhaus S, Leijten P, Jochim J, Melendez-Torres GJ, Gardner F. Effects over time of parenting interventions to reduce physical and emotional violence against children: A systematic review and meta-analysis. *EClinicalMedicine*. 2023;60:102003. doi:10.1016/j.eclinm.2023.102003
