# Supplementary material for: Have parenting programs for disruptive child behavior become less effective?
Source: J Child Psychol Psychiatry. 2025 Sep 18;67(1):127–37. doi: 10.1111/jcpp.70049 (PMC12699127; doi:10.1111/jcpp.70049)
Supplement: Supplementary file 2 — Appendix S2. Included trials. Have parenting programs for disruptive child behavior become less effective? (Leijten et al., 2025; Journal of Child Psychology and Psychiatry). [file JCPP-67-127-s003.docx]

**Appendix II: Included Trials**

Have Parenting Programs for Disruptive Child Behavior Become Less Effective?

(Leijten et al., 2025; *Journal of Child Psychology and Psychiatry*)

| **Lead Author** | **Year** | **Program** | **Country** | ***N*** |
| --- | --- | --- | --- | --- |
|  |  |  |  |  |
| Abikoff | 2015 | New Forest Parenting Programme | USA | 164 |
| Abrahamse | 2021 | Parent-Child Interaction Therapy | Netherlands | 20 |
| Altafim | 2019 | ACT Raising Safe Kids | Brazil | 81 |
| Anastopoulos | 1993 | Barkley's Defiant Children | USA | 34 |
| Au | 2014 | Triple P | Hong Kong | 17 |
| Axberg | 2012 | Incredible Years | Sweden | 62 |
| Azevedo | 2014 | Incredible Years | Portugal | 125 |
| Bagner | 2010 | Parent-Child Interaction Therapy | USA | 28 |
| Bailey | 2012 | 1-2-3 Magic | Australia | 14 |
| Bailey | 2015 | 1-2-3 Magic | Australia | 22 |
| Baker | 2017 | Triple P | Australia | 200 |
| Bakermans-Kranenburg | 2008 | VIPP-SD | Netherlands | 157 |
| Barkley | 2000 | Barkley's Defiant Children | USA | 81 |
| Barone | 2019 | VIPP-SD | Italy | 83 |
| Behan | 2001 | Parents Plus Programme | Ireland | 26 |
| Bernal | 1980 | Behavioural Parent Training | USA | 36 |
| Bjørknes | 2013 | PMTO | Norway | 96 |
| Bjørseth | 2016 | Parent-Child Interaction Therapy | Norway | 81 |
| Bodenmann | 2008 | Triple P | Switzerland | 200 |
| Bradley | 2003 | 1-2-3 Magic | Canada | 198 |
| Braet | 2009 | Parent Management Training | Belgium | 64 |
| Breitenstein | 2012 | Chicago Parent Program | USA | 504 |
| Breitenstein | 2016 | Chicago Parent Program | USA | 79 |
| Breitenstein | 2021 | Chicago Parent Program | USA | 287 |
| Brestan | 1997 | Parent-Child Interaction Therapy | USA | 30 |
| Brock | 2015 | Child-oriented play group | USA | 186 |
| Brotman | 2003 | Incredible Years | USA | 30 |
| Brotman | 2005 | Incredible Years | USA | 99 |
| Carta | 2013 | Planned Activities Training (PAT) | USA | 371 |
| Chacko | 2009 | Strategies to Enhance Positive Parenting | USA | 120 |
| Chacko | 2018 | Caring in Chaos | Denmark | 161 |
| Chan | 2016 | Triple P | Hong Kong | 89 |
| Chan | 2024 | Incorporating emotion coaching into behavioral parent training program | Hong Kong | 119 |
| Chesterfield | 2021 | 1-2-3 Magic | Australia | 60 |
| Coard | 2007 | Black Parenting Strengths and Strategies | USA | 30 |
| Connell | 1997 | Triple P | Australia | 24 |
| Cunningham | 1995 | Parent Training | Canada | 150 |
| Daley | 2021 | New Forest Parenting Programme | UK | 52 |
| David | 2014 | Cognitive behavioral program with emotion-regulation strategies | Romania | 130 |
| Day | 2012 | Empowering Parents, Empowering Communities | UK | 116 |
| Day | 2018 | Triple P | Australia | 183 |
| Day | 2020 | Helping Families Programme | UK | 48 |
| DeGarmo | 2019 | PMTO | USA | 426 |
| De Jong | 2023 | Behavioral Parent Training—Groningen | Netherlands | 110 |
| DeRosier | 2007 | Social Skills Group Intervention Parent Guide | USA | 42 |
| Dishion | 2008 | Family Check-up | USA | 731 |
| Dittman | 2016 | Triple P | New Zealand | 85 |
| Dose | 2017 | Telephone-Assisted Self-Help Intervention (TASH) | Germany | 103 |
| Douglas | 2019 | Solihull Approach | UK | 249 |
| Duncombe | 2016 | Triple P | Australia | 320 |
| DuPaul | 2018 | Behavioral Parent Training | USA | 32 |
| Eichelberger | 2010 | Triple P | Germany | 93 |
| Enebrink | 2012 | Comet | Sweden | 104 |
| Entenberg | 2023 | The Parenting Chatbot Micro Intervention | Argentina | 170 |
| Eşkisu | 2021 | Parenting support program | Turkey | 18 |
| Eyberg | 1995 | Parent-Child Interaction Therapy | USA | 13 |
| Fabiano | 2012 | COACHES | USA | 55 |
| Fanning | 2007 | Success in Parenting Preschoolers | USA | 28 |
| Feng | 2018 | Parent behavioral management skills group | China | 57 |
| Flaherty | 2010 | 1-2-3 Magic | Australia | 38 |
| Foley | 2016 | Parent-Child Interaction Therapy | USA | 44 |
| Forehand | 2010 | Parenting the Strong-Willed Child | USA | 52 |
| Forehand | 2011 | Parenting the Strong-Willed Child | USA | 39 |
| Forgatch | 1979 | PMTO | USA | 12 |
| Forgatch | 1999 | PMTO | USA | 238 |
| Foskolos | 2023 | Triple P | Greece | 124 |
| Francis | 2021 | The Irie Homes Toolbox | Jamaica | 223 |
| Frank | 2015 | Triple P | New Zealand | 42 |
| Franke | 2020 | Triple P | New Zealand | 53 |
| Fung | 2014 | Early Pathways | USA | 137 |
| Gallart | 2005 | Triple P | Australia | 33 |
| Gardner | 2006 | Incredible Years | UK | 76 |
| Gottfredson | 2006 | Strengthening Washington DC Families Project | USA | 715 |
| Gross | 1995 | Incredible Years | USA | 46 |
| Gross | 2003 | Incredible Years | USA | 208 |
| Gross | 2009 | Chicago Parent Program | USA | 253 |
| Hahlweg | 2008 | Triple P | Germany | 69 |
| Hahlweg | 2010 | Triple P | Germany | 186 |
| Hamilton | 1984 | Hanf parent-training model | USA | 18 |
| Hand | 2013 | Parents Plus Programme | Ireland | 75 |
| Hanisch | 2010 | Indicated Prevention Programme for Externalizing Problem Behaviour | Germany | 155 |
| Harris | 2015 | Early Pathways | USA | 199 |
| Haslam | 2013 | Triple P | Australia | 107 |
| Helfenbaum-Kun | 2007 | Incredible Years | USA | 39 |
| Herbert | 2013 | Parenting Your Hyperactive Preschooler | USA | 31 |
| Heubeck | 2023 | Kompetenztraining für Eltern sozial auffä̈lliger Kinder | Germany | 159 |
| Hoang | 2022 | Triple P | Vietnam | 100 |
| Hoath | 2002 | Triple P | Australia | 21 |
| Hornstra | 2021 | Antedecent/Consequent-based behavioural parent training | Netherlands | 62 |
| Hutchings | 2007 | Incredible Years | UK | 153 |
| Jalali | 2009 | Triple P | Iran | 30 |
| Javier | 2016 | The Filipino Family Initiative | USA | 28 |
| Joachim | 2010 | Triple P | Australia | 46 |
| Jones | 2017 | Triple P | UK | 97 |
| Karjalainen | 2019 | Incredible Years | Finland | 102 |
| Keown | 2018 | Triple P | New Zealand | 70 |
| Khademi | 2019 | Triple P | Iran | 94 |
| Kierfeld | 2013 | Bibliographic Intervention | Germany | 48 |
| Kim | 2008 | Incredible Years | USA | 33 |
| Kirby | 2014 | Triple P | Australia | 54 |
| Kjøbli | 2012 | PMTO | Norway | 216 |
| Kjøbli | 2013 | PMTO | Norway | 137 |
| Kleefman | 2014 | Triple P | Netherlands | 209 |
| Kling | 2010 | Comet | Sweden | 101 |
| Lachman | 2017 | Parenting for Lifelong Health | South Africa | 68 |
| Lange | 2018 | New Forest Parenting Programme | Denmark | 164 |
| Larsson | 2009 | Incredible Years | Norway | 81 |
| Lau | 2011 | Incredible Years | USA | 54 |
| Lavigne | 2008 | Incredible Years | USA | 117 |
| Leckey | 2019 | Incredible Years | Ireland | 33 |
| Lefever | 2017 | SafeCare | USA | 229 |
| Leijten | 2016 | Positive Notes | Netherlands | 90 |
| Leijten | 2017 | Incredible Years | Netherlands | 154 |
| Lester | 2015 | Positive Parenting Skills Training Programme | South Africa | 80 |
| Leung | 2003 | Triple P | Hong Kong | 91 |
| Leung | 2013 | Triple P | Hong Kong | 81 |
| Leung | 2014 | Triple P | Hong Kong | 56 |
| Leung | 2015 | Parent-Child Interaction Therapy | Hong Kong | 111 |
| Leung | 2017a | HOPE-20 | Hong Kong | 173 |
| Leung | 2017b | Parent-Child Interaction Therapy | Hong Kong | 64 |
| Leung | 2020 | Happy Parenting Program | China | 154 |
| Little | 2012 | Incredible Years | UK | 161 |
|  |  | Triple P | UK | 146 |
| Long | 1993 | Parent training | USA | 32 |
| Maaskant | 2016 | PMTO | Netherlands | 88 |
| MacKenzie | 2000 | Computer-Assisted Parenting Program | USA | 46 |
| Magen | 1994 | Behavioural skills training and general problem-solving skills training | USA | 56 |
| Maguin | 1994 | PMTO | USA | 104 |
| Malti | 2011 | Triple P | Switzerland | 695 |
| Markie-Dadds | 2006a | Triple P | Australia | 27 |
| Markie-Dadds | 2006b | Triple P | Australia | 63 |
| Martin | 2003 | Triple P | Australia | 42 |
| Mast | 2014 | I-InTERACT | USA | 7 |
| Matalon | 2023 | Positive Parenting for Oppositional Preschoolers (PPOP) | Israel | 85 |
| Matos | 2009 | Parent-Child Interaction Therapy | Puerto Rico | 32 |
| Matsumoto | 2007 | Triple P | Australia | 50 |
| Matsumoto | 2010 | Triple P | Japan | 54 |
| McCabe | 2009 | Parent-Child Interaction Therapy | USA | 58 |
| McGilloway | 2012 | Incredible Years | Ireland | 149 |
| McTaggart | 2003 | Triple P | Australia | 984 |
| Mejía | 2015 | Triple P | Panama | 108 |
| Menting | 2014 | Incredible Years | Netherlands | 133 |
| Mersky | 2016 | Parent-Child Interaction Therapy | USA | 52 |
| Morawska | 2006 | Triple P | Australia | 82 |
| Morawska | 2009 | Triple P | Australia | 75 |
| Morawska | 2011 | Triple P | Australia | 67 |
| Morawska | 2014 | Triple P | Australia | 139 |
| Mullin | 1994 | Parent training | Ireland | 79 |
| Niccols | 2009 | Coping Modeling Problem Solving Approach | Canada | 79 |
| Nicholson | 1999 | Triple P | USA | 26 |
| Nicholson | 2002 | STAR parenting programme | USA | 39 |
| Nixon | 2001 | Parent-Child Interaction Therapy | Australia | 63 |
| Nixon | 2003 | Parent-Child Interaction Therapy | Australia | 34 |
| Nobel | 2020 | Behavioral Parent Training—Groningen | Netherlands | 50 |
| Nogueira | 2021 | Triple P | Portugal | 134 |
| Ogden | 2008 | PMTO | Norway | 112 |
| Olthuis | 2018 | Strongest Families | Canada | 172 |
| Öztürk | 2019 | Triple P | Turkey | 60 |
| Paiva | 2024 | Parent Management Training | Brazil | 38 |
| Palmer | 2023 | Parent Positive | UK | 646 |
| Parra-Cardona | 2017 | PMTO | USA | 67 |
| Patterson | 2002 | Incredible Years | UK | 116 |
| Patterson | 1982 | PMTO | USA | 19 |
| Perrin | 2014 | Incredible Years | USA | 273 |
| Pickering | 2023 | Triple P | Australia | 66 |
| Pisterman | 1989 | Parent training program aimed at improving child compliance | Canada | 46 |
| Pisterman | 1992 | Supporting Father Involvement | Canada | 57 |
| Porzig-Drummond | 2014 | 1-2-3 Magic | Australia | 84 |
| Porzig-Drummond | 2015 | 1-2-3 Magic | Australia | 84 |
| Reedtz | 2011 | Incredible Years | Norway | 186 |
| Rincón | 2018 | Day by Day Program | Chile | 332 |
| Roshan | 2017 | Parent-Child Interaction Therapy | Iran | 59 |
| Sampaio | 2015 | Triple P | Sweden | 355 |
| Sanders | 2000a | Triple P | Australia | 153 |
| Sanders | 2000b | Triple P | Australia | 56 |
| Sanders | 2011 | Triple P | Australia | 121 |
| Sanders | 2012 | Triple P | Australia | 116 |
| Santini | 2017 | Projeto Parceria | Brazil | 40 |
| Sayger | 1988 | PMTO | USA | 43 |
| Scavenius | 2020 | PMTO | Denmark | 127 |
| Schappin | 2013 | Triple P | Netherlands | 67 |
| Schilling | 2017 | CARE | USA | 120 |
| Schuhmann | 1998 | Parent-Child Interaction Therapy | USA | 64 |
| Scott | 2010a | Incredible Years | UK | 112 |
| Scott | 2010b | Incredible Years | UK | 174 |
| Scott | 2014 | Incredible Years | UK | 104 |
| Shaw | 2006 | Family Check-up | USA | 120 |
| Sheeber | 1994 | Temperament-focused psychoeducational intervention | USA | 40 |
| Shimabukuro | 2020 | New Forest Parenting Programme | Japan | 52 |
| Sim | 2014 | Parents Make the Difference | Liberia | 270 |
| Skowron | 2024 | Parent-Child Interaction Therapy | USA | 204 |
| Smallegange | 2016 | Triple P | Netherlands | 105 |
| Smith | 2018 | Triple P | USA | 215 |
| Solís-Cámara | 2004 | Programa de Crianza Estandarizado | Mexico | 40 |
| Solís-Cámara | 2015 | Programa de Crianza Estandarizado | Mexico | 60 |
| Somech | 2012 | Hitkashrut | Israel | 209 |
| Sonuga-Barke | 2001 | New Forest Parenting Programme, Incredible Years | UK | 50 |
| Sonuga-Barke | 2018 | New Forest Parenting Programme | UK | 176 |
| Sourander | 2016 | Strongest Families | Finland | 464 |
| Spaccarelli | 1992 | Incredible Years | USA | 37 |
| Spijkers | 2013 | Triple P | Netherlands | 67 |
| Stallman | 2014 | Triple P | Australia | 204 |
| Stattin | 2015 | Comet, Incredible Years, Cope | Sweden | 361 |
| Stolk | 2008 | VIPP-SD | Netherlands | 237 |
| Strayhorn | 1989 | Training Exercises for Parents of Preschoolers | USA | 98 |
| Sumargi | 2015 | Triple P | Indonesia | 143 |
| Sutton | 1992 | Behavioral parent training | UK | 37 |
| Swift | 2009 | Barkley's Defiant Children | Australia | 29 |
| Sze-To | unpub | Happy Parenting Program | Hong Kong | 242 |
| Taylor | 1998 | Incredible Years | Canada | 36 |
| Thomas | 2011 | Parent-Child Interaction Therapy | Australia | 150 |
| Thompson | 2009 | New Forest Parenting Programme | UK | 41 |
| Tiwawatpakorn | 2022 | Parental Training Program for ADHD Children | Thailand | 79 |
| Tully | 2017 | Triple P | Australia | 45 |
| Tuntipuchitanon | 2024 | Online Positive Parenting Programme | Thailand | 103 |
| Turner | 2006 | Triple P | Australia | 30 |
| Turner | 2007 | Triple P | Australia | 51 |
| Van Aar | 2021 | Positive Notes | Netherlands | 77 |
| Van den Hoofdakker | 2007 | Behavioral Parent Training—Groningen | Netherlands | 96 |
| Van Zeijl | 2006 | VIPP-SD | Netherlands | 237 |
| Vardanian | 2020 | Family groups | USA | 320 |
| Wahler | 1997 | Parent training | USA | 24 |
| Walg | 2012 | Behavioral parent training | Germany | 43 |
| Ward | 2020 | Parenting for Lifelong Health | South Africa | 296 |
| Webster-Stratton | 1982 | Incredible Years | USA | 35 |
| Webster-Stratton | 1984 | Incredible Years | USA | 25 |
| Webster-Stratton | 1988 | Incredible Years | USA | 40 |
| Webster-Stratton | 1990 | Incredible Years | USA | 19 |
| Webster-Stratton | 1992 | Incredible Years | USA | 100 |
| Webster-Stratton | 1997 | Incredible Years | USA | 43 |
| Webster-Stratton | 2004 | Incredible Years | USA | 56 |
| Weeland | 2017 | Incredible Years | Netherlands | 387 |
| Westwood | 2021 | Catch them being good | UK | 58 |
| Wiggins | 2009 | Triple P | Australia | 60 |
| Williams | 2020 | Enhancing Parenting Skills Programme | UK | 58 |
| Williamson | 2014 | Madres a Madres | USA | 194 |
| Wolfe | 1988 | Parent training | Canada | 53 |
| Wood | 2014 | CARE | USA | 120 |
| Wood | 2021 | Child Adult Relationship Enhancement in Primary Care (PriCARE) | USA | 174 |
| Wu | 2024 | Mixed-Mode Parenting Training Program | Hong Kong | 139 |
| Yao | 2022 | Behavioral parent training | Japan | 30 |
| Zangwill | 1983 | Hanf-based parent training model | USA | 15 |
| Zargainejad | unpu | Triple P | Iran | 30 |
| Zhang | 2018 | Behavioral Family Therapy | China | 22 |

**References**

Abikoff, H. B., Thompson, M., Laver‐Bradbury, C., Long, N., Forehand, R. L., Miller Brotman, L., Klein, R. G., Reiss, P, Huo, L., & Sonuga‐Barke, E. (2015). Parent training for preschool ADHD: A randomized controlled trial of specialized and generic programs. *Journal of Child Psychology and Psychiatry, 56*(6), 618−631. https://doi.org/10.1111/jcpp.12346

Abrahamse, M. E., Tsang, V. M., & Lindauer, R. J. (2021). Home-based Parent–Child Interaction Therapy to prevent child maltreatment: a randomized controlled trial. *International Journal of Environmental Research and Public Health, 18*(16), 8244. https://doi.org/10.3390/ijerph18168244

Altafim, E. R. P., & Linhares, M. B. M. (2019). Preventive intervention for strengthening effective parenting practices: A randomized controlled trial. *Journal of Applied Developmental Psychology, 62*, 160–172. https://doi.org/10.1016/j.appdev.2019.03.003

Anastopoulos, A. D., Shelton, T. L., DuPaul, G. J., & Guevremont, D. C. (1993). Parent training for attention-deficit hyperactivity disorder: Its impact on parent functioning. *Journal of Abnormal Child Psychology, 21*(5), 581–596. https://doi.org/10.1007/BF00916320

Au, A., Lau, K. M., Wong, A. H. C., Lam, C., Leung, C., Lau, J., & Lee, Y. K. (2014). The efficacy of a Group Triple P (Positive Parenting Program) for Chinese parents with a child diagnosed with ADHD in Hong Kong: A pilot randomised controlled study. *Australian Psychologist, 49*(3), 151–162. https://doi.org/10.1111/ap.12053

Axberg, U., & Broberg, A. G. (2012). The Incredible Years: Evaluation of the Webster Stratton parent management training programme in Sweden. *Scandinavian Journal of Psychology, 53*(3), 224–232. https://doi.org/10.1111/j.1467-9450.2012.00955.x

Azevedo, A. F., Seabra-Santos, M. J., Gaspar, M. F., & Homem, T. (2014). A parent-based intervention programme involving preschoolers with AD/HD behaviours: Are children's and mothers' effects sustained over time? *European Child and Adolescent Psychiatry, 23*(6), 437–450. https://doi.org/10.1007/s00787-013-0470-2

Bagner, D. M., Sheinkopf, S. J., Vohr, B. R., Lester, B. M. (2010). Parenting intervention for externalizing behavior problems in children born premature: An initial examination. *Journal of Developmental & Behavioral Pediatrics, 31*(3), 209–216. https://doi.org/10.1097/DBP.0b013e3181d5a294

Bailey, E. L., Van der Zwan, R., Phelan, T. W., & Brooks, A. (2012). The 1-2-3 Magic Program: Implementation outcomes of an Australian pilot evaluation with school-aged children. *Child & Family Behavior Therapy, 34*(1), 53–69. https://doi.org/10.1080/07317107.2012.654455

Bailey, E. L., Van der Zwan, R., Phelan, T. W., & Brooks, A. (2015). Keeping it going: evidence of long-term improvements after implementation of the 1-2-3 Magic Parenting Program. *Child & Family Behavior Therapy, 37*(4), 303–320. https://doi.org/10.1080/07317107.2015.1104776

Baker, S., Sanders, M. R., Turner, K. M., & Morawska, A. (2017). A randomized controlled trial evaluating a low-intensity interactive online parenting intervention, Triple P Online Brief, with parents of children with early onset conduct problems. *Behaviour Research and Therapy, 91*, 78–90. https://doi.org/10.1016/j.brat.2017.01.016

Bakermans-Kranenburg, M. J., Van IJzendoorn, M. H., Pijlman, F. T., Mesman, J., & Juffer, F. (2008). Experimental evidence for differential susceptibility: Dopamine D4 receptor polymorphism (DRD4 VNTR) moderates intervention effects on toddlers' externalizing behavior in a randomized controlled trial. *Developmental Psychology, 44*(1), 293–300. https://doi.org/10.1037/0012-1649.44.1.293

Barkley, R. A., Shelton, T. L., Crosswait, C., Moorehouse, M., Fletcher, K., Barrett, S., Jenkins, & Metevia, L. (2000). Multi-method psycho-educational intervention for preschool children with disruptive behavior: Preliminary results at post-treatment. *Journal of Child Psychology and Psychiatry, 41*(3), 319–332. https://doi.org/10.1111/1469-7610.00616

Barone, L., Ozturk, Y., & Lionetti, F. (2019). The key role of positive parenting and children’s temperament in post‐institutionalized children’s socio‐emotional adjustment after adoption placement. A RCT study. *Social Development, 28*(1), 136–151. https://doi.org/10.1111/sode.12329

Behan, J., Fitzpatrick, C., Sharry, J., Carr, A., & Waldron, B. (2001). Evaluation of the Parenting Plus Programme. *Irish Journal of Psychology, 22*(3–4), 238–256. https://doi.org/10.1080/03033910.2001.10558283

Bernal, M. E., Klinnert, M. D., & Schultz, L. A. (1980). Outcome evaluation of behavioral parent training and client‐centered parent counseling for children with conduct problems. *Journal of Applied Behavior Analysis, 13*(4), 677–691. https://doi.org/10.1901/jaba.1980.13-677

Bjørknes, R., & Manger, T. (2013). Can parent training alter parent practice and reduce conduct problems in ethnic minority children? A randomized controlled trial. *Prevention Science, 14*(1), 52–63. https://doi.org/10.1007/s11121-012-0299-9

Bjørseth, Å., & Wichstrøm, L. (2016). Effectiveness of parent-child interaction therapy (PCIT) in the treatment of young children’s behavior problems. A randomized controlled study. *PLOS ONE, 11*, e0159845. https://doi.org/10.1371/journal.pone.0159845

Bodenmann, G., Cina, A., Ledermann, T., & Sanders, M. R. (2008). The efficacy of the Triple P-Positive Parenting Program in improving parenting and child behavior: A comparison with two other treatment conditions. *Behaviour Research and Therapy, 46*, 411–427. https://doi.org/10.1016/j.brat.2008.01.001

Bradley, S. J., Jadaa, D. A., Brody, J., Landy, S., Tallett, S. E., Watson, W., Shea, B., & Stephens, D. (2003). Brief psychoeducational parenting program: An evaluation and 1-year follow-up. *Journal of the American Academy of Child & Adolescent Psychiatry, 42*(10), 1171–1178. https://doi.org/10.1097/00004583-200310000-00007

Braet, C., Meerschaert, T., Merlevede, E., Bosmans, G., Van Leeuwen, K., & De Mey, W. (2009). Prevention of antisocial behaviour: Evaluation of an early intervention programme. *European Journal of Developmental Psychology, 6*(2), 223–240. https://doi.org/10.1080/17405620601033194

Breitenstein, S. M., Fehrenbacher, C., Holod, A. F., & Schoeny, M. E. (2021). A randomized trial of digitally delivered, self-administered parent training in primary care: Effects on parenting and child behavior. *The Journal of Pediatrics, 231*, 207–214. https://doi.org/10.1016/j.jpeds.2020.12.016

Breitenstein, S. M., Fogg, L., Ocampo, E. V., Acosta, D. I., & Gross, D. (2016). Parent use and efficacy of a self-administered, tablet-based parent training intervention: A randomized controlled trial. *JMIR mHealth and uHealth, 4*(2), e5202. https://doi.org/10.2196/mhealth.5202

Breitenstein, S. M., Gross, D., Fogg, L., Ridge, A., Garvey, C., Julion, W., & Tucker, S. (2012). The Chicago Parent Program: Comparing 1‐year outcomes for African American and Latino parents of young children. *Research in Nursing & Health, 35*(5), 475–489. https://doi.org/10.1002/nur.21489

Brestan, E. V., Eyberg, S. M., Boggs, S. R., & Algina, J. (1997). Parent-Child Interaction Therapy: Parents’ perceptions of untreated siblings. *Child and Family Behavior Therapy, 19*(1), 13–28. https://doi.org/10.1300/J019v19n03_02

Brock, R. L., Kochanska, G., O’Hara, M. W., & Grekin, R. S. (2015). Life satisfaction moderates the effectiveness of a play-based parenting intervention in low-income mothers and toddlers. *Journal of Abnormal Child Psychology, 43*(7), 1283–1294. https://doi.org/10.1007/s10802-015-0014-y

Brotman, L. M., Gouley, K. K., Chesir-Teran, D., Dennis, T., Klein, R. G., & Shrout, P. (2005). Prevention for preschoolers at high risk for conduct problems: Immediate outcomes on parenting practices and child social competence. *Journal of Clinical Child and Adolescent Psychology, 34*(4), 724–734. https://doi.org/10.1207/s15374424jccp3404_14

Brotman, L. M., Klein, R. G., Kamboukos, D., Brown, E. J., Coard, S. I., & Sosinsky, L. S. (2003). Preventive intervention for urban, low-income preschoolers at familial risk for conduct problems: A randomized pilot study. *Journal of Clinical Child and Adolescent Psychology, 32*(2), 246–257. https://doi.org/10.1207/S15374424JCCP3202_10

Carta, J. J., Lefever, J. B., Bigelow, K., Borkowski, J., & Warren, S. F. (2013). Randomized trial of a cellular phone-enhanced home visitation parenting intervention. *Pediatrics*, *132*(Supplement_2), S167–S173. https://doi.org/10.1542/peds.2013-1021q

Chacko, A., Fabiano, G. A., Doctoroff, G. L., & Fortson, B. (2018b). Engaging fathers in effective parenting for preschool children using shared book reading: A randomized controlled trial. *Journal of Clinical Child & Adolescent Psychology, 47*(1), 79–93. https://doi.org/10.1080/15374416.2016.1266648

Chacko, A., & Scavenius, C. (2018a). Bending the curve: A community-based behavioral parent training model to address ADHD-related concerns in the voluntary sector in Denmark. *Journal of Abnormal Child Psychology, 46*(3), 505–517. https://doi.org/10.1007/s10802-017-0310-9

Chacko, A., Wymbs, B. T., Wymbs, F. A., Pelham, W. E., Swanger-Gagne, M. S., Girio, E., Pirvics, L., Herbst, L., Guzzo, J., Philips, C., & O'Connor, B. (2009). Enhancing traditional behavioral parent training for single mothers of children with ADHD. *Journal of Clinical Child & Adolescent Psychology, 38*(2), 206–218. https://doi.org/10.1080/15374410802698388

Chan, C. K. Y., Fu, K., & Liu, S. K. Y. (2024). Incorporating emotion coaching into behavioral parent training program: evaluation of its effectiveness. *Child Psychiatry & Human Development*, *55*(1), 236–246. https://doi.org/10.1007/s10578-022-01402-y

Chan, S., Leung, C., & Sanders, M. (2016). A randomised controlled trial comparing the effects of directive and non-directive parenting programmes as a universal prevention programme. *Journal of Children's Services, 11*(1), 38–53. https://doi.org/10.1108/JCS-08-2014-0038

Chesterfield, J. A., Porzig-Drummond, R., Stevenson, R. J., & Stevenson, C. S. (2021). Evaluating a brief behavioral parenting program for parents of school-aged children with ADHD. *Parenting, 21*(3), 216–240. https://doi.org/10.1080/15295192.2020.1777783

Coard, S. I., Foy-Watson, S., Zimmer, C., & Wallace, A. (2007). Considering culturally relevant parenting practices in intervention development and adaptation: A randomized controlled trial of the Black Parenting Strengths and Strategies (BPSS) Program. *The Counseling Psychologist, 35*(6), 797–820. https://doi.org/10.1177/0011000007304592

Connell, S., Sanders, M. R., & Markie-Dadds, C. (1997). Self-directed behavioral family intervention for parents of oppositional children in rural and remote areas. *Behavior Modification, 21*(4), 379–408. https://doi.org/10.1177/01454455970214001

Cunningham, C. E., Bremner, R., & Boyle, M. (1995). Large group community‐based parenting programs for families of preschoolers at risk for disruptive behaviour disorders: utilization, cost effectiveness, and outcome. *Journal of Child Psychology and Psychiatry, 36*(7), 1141–1159. https://doi.org/10.1111/j.1469-7610.1995.tb01362.x

Daley, D., Tarver, J., & Sayal, K. (2021). Efficacy of a self‐help parenting intervention for parents of children with attention deficit hyperactivity disorder in adjunct to usual treatment—Small‐scale randomized controlled trial. *Child: Care, Health and Development, 47*(2), 269–280. https://doi.org/10.1111/cch.12825

David, O. A., David, D., & Dobrean, A. (2014). Efficacy of the rational positive parenting program for child externalizing behavior: Can an emotion-regulation enhanced cognitive-behavioral parent program be more effective than a standard one? *Journal of Evidence-Based Psychotherapies, 14*(2), 159–178.

Day, C., Briskman, J., Crawford, M. J., Foote, L., Harris, L., Boadu, J., McCrone, P., McMurran, M., Michelson, D., Moran, P., Mosse, L., Scott, S., Stahl, D., Ramchandani, P., & Weaver, T. (2020). Randomised feasibility trial of the helping families programme-modified: An intensive parenting intervention for parents affected by severe personality difficulties. *BMJ Open, 10*(2), e033637. https://doi.org/10.1136/bmjopen-2019-033637

Day, C., Michelson, D., Thomson, S., Penney, C., & Draper, L. (2012). Evaluation of a peer led parenting intervention for disruptive behaviour problems in children: Community based randomised controlled trial. *British Medical Journal, 344*, e1107. https://doi.org/10.1136/bmj.e1107

Day, J. J., & Sanders, M. R. (2018). Do parents benefit from help when completing a self-guided parenting program online? A randomized controlled trial comparing Triple P Online with and without telephone support. *Behavior Therapy, 49*(6), 1020–1038. https://doi.org/10.1016/j.beth.2018.03.002

DeGarmo, D. S., & Jones, J. A. (2019). Fathering Through Change (FTC) intervention for single fathers: Preventing coercive parenting and child problem behaviors. *Development and Psychopathology, 31*(5), 1801–1811. https://doi.org/10.1017/S0954579419001019

De Jong, S. R., Van den Hoofdakker, B. J., Van der Veen-Mulders, L., Veenman, B., Twisk, J. W., Oosterlaan, J., & Luman, M. (2023). The efficacy of a self-help parenting program for parents of children with externalizing behavior: A randomized controlled trial. *European Child & Adolescent Psychiatry, 32*(10), 2031–2042. https://doi.org/10.1007/s00787-022-02028-0

DeRosier, M. E., & Gilliom, M. (2007). Effectiveness of a parent training program for improving children’s social behavior. *Journal of Child and Family Studies, 16*(5), 660–670. https://doi.org/10.1007/s10826-006-9114-1

Dishion, T. J., Shaw, D., Connell, A., Gardner, F., Weaver, C., & Wilson, M. (2008). The family check‐up with high‐risk indigent families: Preventing problem behavior by increasing parents’ positive behavior support in early childhood. *Child Development, 79*(5), 1395–1414. https://doi.org/10.1111/j.1467-8624.2008.01195.x

Dittman, C. K., Farruggia, S. P., Keown, L. J., & Sanders, M. R. (2016). Dealing with disobedience: An evaluation of a brief parenting intervention for young children showing noncompliant behavior problems. *Child Psychiatry & Human Development, 47*(1), 102–112. https://doi.org/10.1007/s10578-015-0548-9

Dose, C., Hautmann, C., Buerger, M., Schuermann, S., Woitecki, K., & Döpfner, M. (2017). Telephone‐assisted self‐help for parents of children with attention‐deficit/hyperactivity disorder who have residual functional impairment despite methylphenidate treatment: A randomized controlled trial. *Journal of Child Psychology and Psychiatry*, *58*(6), 682–690. https://doi.org/10.1111/jcpp.12661

Douglas, H., & Johnson, R. (2019). The Solihull Approach 10-week programme: a randomised controlled trial. *Community Practitioner, 92*(7), 45–47.

Duncombe, M. E., Havighurst, S. S., Kehoe, C. E., Holland, K. A., Frankling, E. J., & Stargatt, R. (2016). Comparing an emotion-and a behavior-focused parenting program as part of a multsystemic intervention for child conduct problems. *Journal of Clinical Child & Adolescent Psychology, 45*(3), 320–334. https://doi.org/10.1080/15374416.2014.963855

DuPaul, G. J., Kern, L., Belk, G., Custer, B., Daffner, M., Hatfield, A., & Peek, D. (2018). Face-to-face versus online behavioral parent training for young children at risk for ADHD: Treatment engagement and outcomes. *Journal of Clinical Child & Adolescent Psychology, 47*(sup1), S369–S383. https://doi.org/10.1080/15374416.2017.1342544

Eichelberger, I., Plück, J., Hanisch, C., Hautmann, C., Jänen, N., & Döpfner, M. (2010). Effekte universeller Prävention mit dem Gruppenformat des Elterntrainings Triple P auf das kindliche Problemverhalten, das elterliche Erziehungsverhalten und die psychische Belastung der Eltern. *Zeitschrift für klinische Psychologie und Psychotherapie, 39*(1), 24–32. https://doi.org/10.1026/1616-3443/a000006

Enebrink, P., Högström, J., Forster, M., & Ghaderi, A. (2012). Internet-based parent management training: A randomized controlled study. *Behaviour research and therapy, 50*(4), 240–249. https://doi.org/10.1016/j.brat.2012.01.006

Entenberg, G. A., Mizrahi, S., Walker, H., Aghakhani, S., Mostovoy, K., Carre, N., Marshall, Z., Dosovitsky, G., Benfica, D., Rousseau, A., Lin, G., & Bunge, E. L. (2023). AI-based chatbot micro-intervention for parents: Meaningful engagement, learning, and efficacy. *Frontiers in Psychiatry*, *14*. https://doi.org/10.3389/fpsyt.2023.1080770

Eşkisu, M., & Kapçı, E. G. (2021). Efficacy of the parenting support program on child behavior problems. *Scandinavian Journal of Psychology, 62*(4), 449–459. https://doi.org/10.1111/sjop.12726

Eyberg, S. M., Boggs, S. R., & Algina, J. (1995). Parent-child interaction therapy: a psychosocial model for the treatment of young children with conduct problem behavior and their families. *Psychopharmacology Bulletin*, *31*(1), 83–91.

Fabiano, G. A., Pelham, W. E., Cunningham, C. E., Yu, J., Gangloff, B., Buck, M., Linke, S., Gormley, M., & Gera, S. (2012). A waitlist-controlled trial of behavioral parent training for fathers of children with ADHD. *Journal of Clinical Child & Adolescent Psychology, 41*(3), 337–345. https://doi.org/10.1080/15374416.2012.654464

Fanning, J. L. (2007). Parent training for caregivers of typically developing, economically disadvantaged preschoolers: An initial study in enhancing language development, avoiding behavior problems, and regulating family stress. [Doctoral dissertation, University of Oregon]

Feng, Z., Cui, Z., Huang, M., Zhong, Q., Chen, Y., & Wan, G. (2018). Parent behavioral management skills group training to treat preschoolers’ externalizing problem behaviors. *Chinese Journal of Practical Pediatrics, 33*(7), 538–541. https://doi.org/10.19538/j.ek2018070613

Flaherty, R., & Cooper, R. (2010). Piloting a parenting skills program in an Australian rural child protection setting. *Children Australia, 35*(3), 18–24. https://doi.org/10.1017/S1035077200001139

Foley, K., McNeil, C. B., Norman, M., & Wallace, N. M. (2016). Effectiveness of group format Parent-Child Interaction Therapy compared to treatment as usual in a community outreach organization. *Child & Family Behavior Therapy, 38*(4), 279–298. https://doi.org/10.1080/07317107.2016.1238688

Forehand, R. L., Merchant, M. J., Long, N., & Garai, E. (2010). An examination of parenting the strong-willed child as bibliotherapy for parents. *Behavior Modification, 34*(1), 57–76. https://doi.org/10.1177/0145445509356351

Forehand, R. L., Merchant, M. J., Parent, J., Long, N., Linnea, K., & Baer, J. (2011). An examination of a group curriculum for parents of young children with disruptive behavior. *Behavior Modification, 35*(3), 235–251. https://doi.org/10.1177/0145445510393731

Forgatch, M. S., & DeGarmo, D. S. (1999). Parenting through change: an effective prevention program for single mothers*. Journal of Consulting and Clinical Psychology, 67*(5), 711–724. https://doi.org/10.1037/0022-006X.67.5.711

Forgatch, M. S., & Toobert, D. J. (1979). A cost-effective parent training program for use with normal preschool children. *Journal of Pediatric Psychology, 4*(2), 129–145. https://doi.org/10.1093/jpepsy/4.2.129

Foskolos, K., Gardner, F., & Montgomery, P. (2023). Brief parenting seminars for preventing child behavioral and emotional difficulties: A pilot randomized controlled trial. *Journal of Child and Family Studies, 32*(10), 3063–3075. https://doi.org/10.1007/s10826-023-02653-6

Francis, T., & Baker-Henningham, H. (2021). The Irie Homes Toolbox: A cluster randomized controlled trial of an early childhood parenting program to prevent violence against children in Jamaica. *Children and Youth Services Review, 126*, 106060. https://doi.org/10.1016/j.childyouth.2021.106060

Frank, T. J., Keown, L. J., & Sanders, M. R. (2015). Enhancing father engagement and interparental teamwork in an evidence-based parenting intervention: A randomized-controlled trial of outcomes and processes. *Behavior Therapy, 46*(6), 749–763. https://doi.org/10.1016/j.beth.2015.05.008

Franke, N., Keown, L. J., & Sanders, M. R. (2020). An RCT of an online parenting program for parents of preschool-aged children with ADHD symptoms. *Journal of Attention Disorders, 24*(12), 1716–1726. https://doi.org/10.1177/1087054716667598

Fung, M. P., & Fox, R. A. (2014). The culturally-adapted Early Pathways program for young Latino children in poverty: A randomized controlled trial. *Journal of Latina/o Psychology, 2*(3), 131–145. https://doi.org/10.1037/lat0000019

Gallart, S. C., & Matthey, S. (2005). The effectiveness of Group Triple P and the impact of the four telephone contacts. *Behaviour Change, 22*(2), 71–80. https://doi.org/10.1375/bech.2005.22.2.71

Gardner, F., Burton, J., & Klimes, I. (2006). Randomised controlled trial of a parenting intervention in the voluntary sector for reducing child conduct problems: Outcomes and mechanisms of change. *Journal of Child Psychology and Psychiatry, 47*(11), 1123–1132. https://doi.org/10.1111/j.1469-7610.2006.01668.x

Gottfredson, D., Kumpfer, K., Polizzi-Fox, D., Wilson, D., Puryear, V., Beatty, P., & Vilmenay, M. (2006). The Strengthening Washington DC Families Project: A randomized effectiveness trial of family-based prevention. *Prevention Science, 7*(1), 57–74. https://doi.org/10.1007/s11121-005-0017-y

Gross, D., Fogg, L., & Tucker, S. (1995). The efficacy of parent training for promoting positive parent—toddler relationships. *Research in Nursing & Health, 18*, 489–499. https://doi.org/10.1002/nur.4770180605

Gross, D., Fogg, L., Webster-Stratton, C., Garvey, C., Julion, W., & Grady, J. (2003). Parent training of toddlers in day care in low-income urban communities. *Journal of Consulting and Clinical Psychology, 71*(2), 261–278. https://doi.org/10.1037/0022-006X.71.2.261

Gross, D., Garvey, C., Julion, W., Fogg, L., Tucker, S., & Mokros, H. (2009). Efficacy of the Chicago Parent Program with low-income African American and Latino parents of young children. *Prevention Science, 10*(1), 54–65. https://doi.org/10.1007/s11121-008-0116-7

Hahlweg, K., Heinrichs, N., Kuschel, A., Bertram, H., & Naumann, S. (2010). Long-term outcome of a randomized controlled universal prevention trial through a positive parenting program: Is it worth the effort? *Child and Adolescent Psychiatry and Mental Health, 4*, 1–14. https://doi.org/10.1186/1753-2000-4-14

Hahlweg, K., Heinrichs, N., Kuschel, A., & Feldmann, M. (2008). Therapist-assisted, self-administered bibliotherapy to enhance parental competence: Short-and long-term effects. *Behavior Modification, 32*(5), 659–681. https://doi.org/10.1177/0145445508317131

Hamilton, S. B., & MacQuiddy, S. L. (1984). Self‐administered behavioral parent training: Enhancement of treatment efficacy using a time‐out signal seat. *Journal of Clinical Child & Adolescent Psychology, 13*(1), 61–69. https://doi.org/10.1080/15374418409533171

Hand, A., McDonnell, E., Honari, B., & Sharry, J. (2013). A community led approach to delivery of the Parents Plus Children's Programme for the parents of children aged 6–11. *International Journal of Clinical and Health Psychology, 13*(2), 87–90. https://doi.org/10.1016/S1697-2600(13)70011-5

Hanisch, C., Freund-Braier, I., Hautmann, C., Jänen, N., Plück, J., Brix, G., Eichelberger, I., & Döpfner, M. (2010). Detecting effects of the indicated prevention Programme for Externalizing Problem behaviour (PEP) on child symptoms, parenting, and parental quality of life in a randomized controlled trial. *Behavioural and Cognitive Psychotherapy, 38*(1), 95–112. https://doi.org/10.1017/S1352465809990440

Harris, S. E., Fox, R. A., & Love, J. R. (2015). Early pathways therapy for young children in poverty: A randomized controlled trial. *Counseling Outcome Research and Evaluation, 6*(1), 3–17. https://doi.org/10.1177/2150137815573628

Haslam, D. M., Sanders, M. R., & Sofronoff, K. (2013). Reducing work and family conflict in teachers: A randomised controlled trial of Workplace Triple P. *School Mental Health, 5*, 70–82. https://doi.org/10.1007/s12310-012-9091-z

Helfenbaum-Kun, E. D., & Ortiz, C. (2007). Parent-training groups for fathers of head start children: A pilot study of their feasibility and impact on child behavior and intra-familial relationships. *Child & Family Behavior Therapy, 29*(2), 47–64. https://doi.org/10.1300/J019v29n02_04

Herbert, S. D. (2013). Parent training for families of hyperactive preschool-aged children. [Doctoral dissertation, University of Massachusetts Amherst]

Heubeck, B. G., Welvaert, M., & Richardson, A. (2023). Situation‐specific outcomes (Home Situations Questionnaire) in a randomized controlled trial of individual versus group parent training for children with hyperkinetic disorder/attention‐deficit hyperactivity disorder. *British Journal of Clinical Psychology*, *62*(2), 372–391. https://doi.org/10.1111/bjc.12413

Hoang, N.-P. T., Kirby, J. N., Haslam, D. M., & Sanders, M. R. (2022). Promoting positive relationship between parents and arandparents: A randomized controlled trial of Group Triple P plus compassion in Vietnam. *Behavior Therapy*, *53*(6), 1175–1190. https://doi.org/10.1016/j.beth.2022.06.002

Hoath, F. E., & Sanders, M. R. (2002). A feasibility study of Enhanced Group Triple P—Positive Parenting Program for parents of children with attention-deficit/hyperactivity disorder. *Behaviour Change, 19*(4), 191–206. https://doi.org/10.1375/bech.19.4.191

Hornstra, R., Van der Oord, S., Staff, A. I., Hoekstra, P. J., Oosterlaan, J., Van der Veen-Mulders, L., Luman, M., & Van den Hoofdakker, B. J. (2021). Which techniques work in behavioral parent training for children with ADHD? A randomized controlled microtrial. *Journal of Clinical Child & Adolescent Psychology, 50*(6), 888–903. https://doi.org/10.1080/15374416.2021.1955368

Hutchings, J., Bywater, T., Daley, D., Gardner, F., Whitaker, C., Jones, K., Eames, C., & Edwards, R. T. (2007). Parenting intervention in Sure Start services for children at risk of developing conduct disorder: Pragmatic randomised controlled trial. *British Medical Journal, 334*, 678–685. https://doi.org/10.1136/bmj.39126.620799.55

Jalali, M., Shaeeri, M. R., Tahmasian, K., & Pourahmadi, E. (2009). The effects of Triple P-Positive Parenting Program on 7-10 year old children with oppositional defiant disorder (ODD). *Clinical Psychology and Personality, 7*(1), 29–38.

Javier, J. R., Coffey, D. M., Schrager, S. M., Palinkas, L. A., & Miranda, J. (2016). Parenting intervention for prevention of behavioral problems in elementary school-age Filipino-American children: A pilot study in churches. *Journal of Developmental and Behavioral Pediatrics, 37*(9), 737–745. https://doi.org/10.1097/DBP.0000000000000342

Joachim, S., Sanders, M. R., & Turner, K. M. (2010). Reducing preschoolers’ disruptive behavior in public with a brief parent discussion group. *Child Psychiatry & Human Development, 41*(1), 47–60. https://doi.org/10.1007/s10578-009-0151-z

Jones, S. H., Jovanoska, J., Calam, R., Wainwright, L. D., Vincent, H., Asar, O., Diggle, P. J., Parker, R., Long, R., Sanders, M., & Lobban, F. (2017). Web‐based integrated bipolar parenting intervention for parents with bipolar disorder: A randomised controlled pilot trial. *Journal of Child Psychology and Psychiatry, 58*(9), 1033–1041. https://doi.org/10.1111/jcpp.12745

Karjalainen, P., Kiviruusu, O., Aronen, E. T., & Santalahti, P. (2019). Group-based parenting program to improve parenting and children's behavioral problems in families using special services: A randomized controlled trial in a real-life setting. *Children and Youth Services Review, 96*, 420–429. https://doi.org/10.1016/j.childyouth.2018.12.004

Keown, L. J., Sanders, M. R., Franke, N., & Shepherd, M. (2018). Te Whānau Pou Toru: A randomized controlled trial (RCT) of a culturally adapted low-intensity variant of the Triple P-Positive Parenting Program for indigenous Māori families in New Zealand. *Prevention Science, 19*(7), 954–965. https://doi.org/10.1007/s11121-018-0886-5

Khademi, M., Ayatmehr, F., Khosravan Mehr, N., Razjooyan, K., Davari Ashtiani, R., & Arabgol, F. (2019). Evaluation of the effects of positive parenting program on symptoms of preschool children with Attention Deficit Hyperactivity Disorder. *Practice in Clinical Psychology, 7*(1), 11–20. https://doi.org/10.32598/jpcp.7.1.11

Kierfeld, F., Ise, E., Hanisch, C., Görtz-Dorten, A., & Döpfner, M. (2013). Effectiveness of telephone-assisted parent-administered behavioural family intervention for preschool children with externalizing problem behaviour: A randomized controlled trial. *European Child & Adolescent Psychiatry, 22*, 553–565. https://doi.org/10.1007/s00787-013-0397-7

Kim, E., Cain, K. C., & Webster-Stratton, C. (2008). The preliminary effect of a parenting program for Korean American mothers: A randomized controlled experimental study. *International Journal of Nursing Studies, 45*(9), 1261–1273. https://doi.org/10.1016/j.ijnurstu.2007.10.002

Kirby, J. N., & Sanders, M. R. (2014). A randomized controlled trial evaluating a parenting program designed specifically for grandparents. *Behaviour Research and Therapy, 52*(1), 35–44. https://doi.org/10.1016/j.brat.2013.11.002

Kjøbli, J., & Ogden, T. (2012). A randomized effectiveness trial of brief parent training in primary care settings. *Prevention Science, 13*, 616–626. https://doi.org/10.1007/s11121-012-0289-y

Kjøbli, J., Hukkelberg, S., & Ogden, T. (2013). A randomized trial of group parent training: Reducing child conduct problems in real-world settings. *Behaviour Research and Therapy, 51*(3), 113–121. https://doi.org/10.1016/j.brat.2012.11.006

Kleefman, M., Jansen, D. E., Stewart, R. E., & Reijneveld, S. A. (2014). The effectiveness of Stepping Stones Triple P parenting support in parents of children with borderline to mild intellectual disability and psychosocial problems: A randomized controlled trial. *BMC Medicine, 12*(1), 1–10. https://doi.org/10.1186/s12916-014-0191-5

Kling, Å., Forster, M., Sundell, K., & Melin, L. (2010). A randomized controlled effectiveness trial of parent management training with varying degrees of therapist support. *Behavior Therapy, 41*(4), 530–542. https://doi.org/10.1016/j.beth.2010.02.004

Lachman, J. M., Cluver, L., Ward, C. L., Hutchings, J., Mlotshwa, S., Wessels, I., & Gardner, F. (2017). Randomized controlled trial of a parenting program to reduce the risk of child maltreatment in South Africa. *Child Abuse & Neglect, 72*, 338–351. https://doi.org/10.1016/j.chiabu.2017.08.014

Lange, A. M., Daley, D., Frydenberg, M., Houmann, T., Kristensen, L. J., Rask, C., Sonuga-Barke, E., Søndergaard-Baden, S., Udupi, A., & Thomsen, P. H. (2018). Parent training for preschool ADHD in routine, specialist care: A randomized controlled trial. *Journal of the American Academy of Child & Adolescent Psychiatry, 57*(8), 593–602. https://doi.org/10.1016/j.jaac.2018.04.014

Larsson, B., Fossum, S., Clifford, G., Drugli, M. B., Handegård, B. H., & Mørch, W. T. (2009). Treatment of oppositional defiant and conduct problems in young Norwegian children: Results of a randomized controlled trial. *European Child & Adolescent Psychiatry, 18*(6), 42–52. https://doi.org/10.1007/s00787-008-0702-z

Lau, A. S., Fung, J. J., Ho, L. Y., Liu, L. L., & Gudiño, O. G. (2011). Parent training with high-risk immigrant Chinese families: A pilot group randomized trial yielding practice-based evidence. *Behavior Therapy, 42*(3), 413–426. https://doi.org/10.1016/j.beth.2010.11.001

Lavigne, J. V., LeBailly, S. A., Gouze, K. R., Cicchetti, C., Pochyly, J., Arend, R., Jessup, B. W., & Binns, H. J. (2008). Treating oppositional defiant disorder in primary care: a comparison of three models. *Journal of Pediatric Psychology, 33*(5), 449–461. https://doi.org/10.1093/jpepsy/jsm074

Leckey Y, McGilloway S, Hickey G, Bracken-Scally M, Kelly P, Furlong, M. (2019). A randomised control trial of parent and child training programmes (versus wait list control) for children with ADHD-type behaviours: A pilot study. *Child Care in Practice,* *25*(4), 419–438. https://doi.org/10.1080/13575279.2018.1481368

Lefever, J. E. B., Bigelow, K. M., Carta, J. J., Borkowski, J. G., Grandfield, E., McCune, L., Irvin, D. W., & Warren, S. F. (2017). Long-term impact of a cell phone–enhanced parenting intervention. *Child Maltreatment, 22*(4), 305–314. https://doi.org/10.1177/1077559517723125

Leijten, P., Raaijmakers, M. A., Orobio de Castro, B., Van den Ban, E., & Matthys, W. (2017). Effectiveness of the incredible years parenting program for families with socioeconomically disadvantaged and ethnic minority backgrounds. *Journal of Clinical Child & Adolescent Psychology, 46*(1), 59–73. https://doi.org/10.1080/15374416.2015.1038823

Leijten, P., Thomaes, S., Orobio de Castro, B., Dishion, T. J., & Matthys, W. (2016). What good is labeling what's good? A field experimental investigation of parental labeled praise and child compliance. *Behaviour Research and Therapy, 87*, 134–141. https://doi.org/10.1016/j.brat.2016.09.008

Lester, S. N. (2015). *Evaluation of the Parent Centre's positive parenting skills training programme: a randomised controlled trial*. [Master’s thesis, University of Cape Town]. OpenUCT. http://hdl.handle.net/11427/15615

Leung, C., Chan, S., Ip, H. L., Szeto, H., Lee, M., Chan, K., & Chan, M. (2020). Effectiveness of parenting program for Macau shift work parents: Randomized controlled trial. *Research on Social Work Practice, 30*(6), 612–622. https://doi.org/10.1177/1049731520903429

Leung, C., Fan, A., & Sanders, M. R. (2013). The effectiveness of a Group Triple P with Chinese parents who have a child with developmental disabilities: A randomized controlled trial. *Research in Developmental Disabilities, 34*(3), 976–984. https://doi.org/10.1016/j.ridd.2012.11.023

Leung, C., Sanders, M., Fung, B., & Kirby, J. (2014). The effectiveness of the Grandparent Triple P program with Hong Kong Chinese families: A randomized controlled trial. *Journal of Family Studies, 20*(2), 104–117. https://doi.org/10.1080/13229400.2014.11082000

Leung, C., Sanders, M. R., Leung, S., Mak, R., & Lau, J. (2003). An outcome evaluation of the implementation of the Triple P‐Positive Parenting Program in Hong Kong. *Family Process, 42*(4), 531–544. https://doi.org/10.1111/j.1545-5300.2003.00531.x

Leung, C., Tsang, S., & Kwan, H. W. (2017a). Efficacy of a universal parent training program (HOPE-20) cluster randomized controlled trial. *Research on Social Work Practice, 27*(5), 523–537. https://doi.org/10.1177/1049731515593810

Leung, C., Tsang, S., Ng, G. S., & Choi, S. Y. (2017b). Efficacy of parent–child interaction therapy with Chinese ADHD children: Randomized controlled trial. *Research on Social Work Practice, 27*(1), 36–47. https://doi.org/10.1177/1049731516643837

Leung, C., Tsang, S., Sin, T. C., & Choi, S. Y. (2015). The efficacy of parent–child interaction therapy with Chinese families: Randomized controlled trial. *Research on Social Work Practice, 25*(1), 117–128. https://doi.org/10.1177/1049731513519827

Little, M., Berry, V. L., Morpeth, L., Blower, S., Axford, N., Taylor, R., Bywater, T., Lehtonen, M., & Tobin, K. (2012). The impact of three evidence-based programmes delivered in public systems in Birmingham, UK. *International Journal of Conflict and Violence, 6*(2), 260–272. https://doi.org/10.4119/ijcv-2917

Long, N., Rickert, V. I., & Ashcraft, E. W. (1993). Bibliotherapy as an adjunct to stimulant medication in the treatment of attention-deficit hyperactivity disorder. *Journal of Pediatric Health Care, 7*(2), 82–88. https://doi.org/10.1016/0891-5245(93)90078-V

Maaskant, A. M., Van Rooij, F. B., Overbeek, G. J., Oort, F. J., & Hermanns, J. M. (2016). Parent training in foster families with children with behavior problems: Follow-up results from a randomized controlled trial. *Children and Youth Services Review, 70*, 84–94. https://doi.org/10.1016/j.childyouth.2016.09.005

MacKenzie, E. P., & Hilgedick, J. M. (2000). The Computer-Assisted Parenting Program (CAPP): The use of a computerized behavioral parent training program as an educational tool. *Child & Family Behavior Therapy*, *21*(4), 23–43. https://doi.org/10.1300/j019v21n04_02

Magen, R. H., & Rose, S. D. (1994). Parents in groups: Problem solving versus behavioral skills training. *Research on Social Work Practice, 4*(2), 172–191. https://doi.org/10.1177/104973159400400204

Maguin, E., Zucker, R. A., & Fitzgerald, H. E. (1994). The path to alcohol problems through conduct problems: A family-based approach to very early intervention with risk. *Journal of Research on Adolescence, 4*(2), 249–269. https://doi.org/10.1207/s15327795jra0402_5

Malti, T., Ribeaud, D., & Eisner, M. P. (2011). The effectiveness of two universal preventive interventions in reducing children's externalizing behavior: A cluster randomized controlled trial. *Journal of Clinical Child & Adolescent Psychology, 40*(5), 677–692. https://doi.org/10.1080/15374416.2011.597084

Markie-Dadds, C., & Sanders, M. R. (2006a). A controlled evaluation of an enhanced self-directed behavioural family intervention for parents of children with conduct problems in rural and remote areas. *Behaviour Change, 23*(1), 55–72. https://doi.org/10.1375/bech.23.1.55

Markie-Dadds, C., & Sanders, M. R. (2006b). Self-directed Triple P (Positive Parenting Program) for mothers with children at-risk of developing conduct problems. *Behavioural and Cognitive Psychotherapy, 34*(3), 259–275. https://doi.org/10.1017/S1352465806002797

Martin, A. J., & Sanders, M. R. (2003). Balancing work and family: A controlled evaluation of the Triple P‐Positive Parenting Program as a work‐site intervention. *Child and Adolescent Mental Health, 8*(4), 161–169. https://doi.org/10.1111/1475-3588.00066

Mast, J. E., Antonini, T. N., Raj, S. P., Oberjohn, K. S., Cassedy, A., Makoroff, K. L., & Wade, S. L. (2014). Web-based parenting skills to reduce behavior problems following abusive head trauma: A pilot study. *Child Abuse & Neglect, 38*(9), 1487–1495. https://doi.org/10.1016/j.chiabu.2014.04.012

Matalon, C., & Turliuc, M. N. (2023). The effectiveness of a home-based parent management training for preschoolers with oppositional behaviors in Israel: A randomized controlled trial. *Early Childhood Research Quarterly*, *63*, 386–399. https://doi.org/10.1016/j.ecresq.2023.01.010

Matos, M., Bauermeister, J. J., & Bernal, G. (2009). Parent‐child interaction therapy for Puerto Rican preschool children with ADHD and behavior problems: A pilot efficacy study. *Family Process, 48*(2), 232–252. https://doi.org/10.1111/j.1545-5300.2009.01279.x

Matsumoto, Y., Sofronoff, K., & Sanders, M. R. (2007). The efficacy and acceptability of the Triple P-Positive Parenting Program with Japanese parents. *Behaviour Change, 24*(4), 205–218. https://doi.org/10.1375/bech.24.4.205

Matsumoto, Y., Sofronoff, K., & Sanders, M. R. (2010). Investigation of the effectiveness and social validity of the Triple P Positive Parenting Program in Japanese society. *Journal of Family Psychology, 24*(1), 87–91. https://doi.org/10.1037/a0018181

McCabe, K., & Yeh, M. (2009). Parent–child interaction therapy for Mexican Americans: A randomized clinical trial. *Journal of Clinical Child & Adolescent Psychology, 38*(5), 753–759. https://doi.org/10.1080/15374410903103544

McGilloway, S., Mhaille, G. N., Bywater, T., Furlong, M., Leckey, Y., Kelly, P., Comiskey, C., & Donnelly, M. (2012). A parenting intervention for childhood behavioral problems: A randomized controlled trial in disadvantaged community-based settings. *Journal of Consulting and Clinical Psychology, 80*(1), 116–127. https://doi.org/10.1037/a0026304

McTaggart, P., & Sanders, M. R. (2003). The transition to school project: Results from the classroom. *The Australian e-Journal for the Advancement of Mental Health*, *2*(3), 144–155. https://doi.org/10.5172/jamh.2.3.144

Mejía, A., Calam, R., & Sanders, M. R. (2015). A pilot randomized controlled trial of a brief parenting intervention in low-resource settings in Panama. *Prevention Science, 16*(2), 707–717. https://doi.org/10.1007/s11121-015-0551-1

Menting, A. T., de Castro, B. O., Wijngaards-de Meij, L. D., & Matthys, W. (2014). A trial of parent training for mothers being released from incarceration and their children. *Journal of Clinical Child & Adolescent Psychology, 43*(3), 381–396. https://doi.org/10.1080/15374416.2013.817310

Mersky, J. P., Topitzes, J., Grant-Savela, S. D., Brondino, M. J., & McNeil, C. B. (2016). Adapting parent–child interaction therapy to foster care: Outcomes from a randomized trial. *Research on Social Work Practice, 26*(2), 157–167. https://doi.org/10.1177/1049731514543023

Morawska, A., Haslam, D., Milne, D., & Sanders, M. R. (2011). Evaluation of a brief parenting discussion group for parents of young children. *Journal of Developmental & Behavioral Pediatrics, 32*(2), 136–145. https://doi.org/10.1097/DBP.0b013e3181f17a28

Morawska, A., & Sanders, M. R. (2006). Self-administered behavioral family intervention for parents of toddlers: Part I. Efficacy. *Journal of Consulting and Clinical Psychology, 74*(1), 10–19. https://doi.org/10.1037/0022-006X.74.1.10

Morawska, A., & Sanders, M. (2009). An evaluation of a behavioural parenting intervention for parents of gifted children. *Behaviour Research and Therapy, 47*(6), 463–470. https://doi.org/10.1016/j.brat.2009.02.008

Morawska, A., Tometzki, H., & Sanders, M. R. (2014). An evaluation of the efficacy of a Triple P-Positive Parenting Program podcast series. *Journal of Developmental & Behavioral Pediatrics, 35*(2), 128–137. https://doi.org/10.1097/DBP.0000000000000020

Mullin, E., Quigley, K., & Glanville, B. (1994). A controlled evaluation of the impact of a parent training programme on child behaviour and mothers' general well-being. *Counselling Psychology Quarterly, 7*(2), 167–180. https://doi.org/10.1080/09515079408254143

Niccols, A. (2009). Immediate and short‐term outcomes of the ‘COPEing with Toddler Behaviour’ parent group. *Journal of Child Psychology and Psychiatry, 50*(5), 617–626. https://doi.org/10.1111/j.1469-7610.2008.02007.x

Nicholson, B., Anderson, M., Fox, R., & Brenner, V. (2002). One family at a time: A prevention program for at‐risk parents. *Journal of Counseling & Development, 80*(3), 362–371. https://doi.org/10.1002/j.1556-6678.2002.tb00201.x

Nicholson, J. M., & Sanders, M. R. (1999). Randomized controlled trial of behavioral family intervention for the treatment of child behavior problems in stepfamilies. *Journal of Divorce & Remarriage, 30*(3-4), 1–23. https://doi.org/10.1300/J087v30n03_01

Nixon, R. D. (2001). Changes in hyperactivity and temperament in behaviourally disturbed preschoolers after parent–child interaction therapy (PCIT). *Behaviour Change, 18*(3), 168–176. https://doi.org/10.1375/bech.18.3.168

Nixon, R. D., Sweeney, L., Erickson, D. B., & Touyz, S. W. (2003). Parent-child interaction therapy: a comparison of standard and abbreviated treatments for oppositional defiant preschoolers. *Journal of Consulting and Clinical Psychology, 71*(2), 251–260. https://doi.org/10.1037/0022-006X.71.2.251

Nobel, E., Hoekstra, P. J., Brunnekreef, J. A., Messink-de Vries, D. E., Fischer, B., Emmelkamp, P. M., & Van den Hoofdakker, B. J. (2020). Home-based parent training for school-aged children with attention-deficit/hyperactivity disorder and behavior problems with remaining impairing disruptive behaviors after routine treatment: A randomized controlled trial. *European Child & Adolescent Psychiatry, 29*(3), 395–408. https://doi.org/10.1007/s00787-019-01375-9

Nogueira, S., Abreu-Lima, I., Canário, C., & Cruz, O. (2021). Group Triple P–A randomized controlled trial with low-income mothers. *Children and Youth Services Review, 121*, 105862. https://doi.org/10.1016/j.childyouth.2020.105862

Ogden, T., & Hagen, K. A. (2008). Treatment effectiveness of parent management training in Norway: A randomized controlled trial of children with conduct problems. *Journal of Consulting and Clinical Psychology, 76*(4), 607–621. https://doi.org/10.1037/0022-006X.76.4.607

Olthuis, J. V., McGrath, P. J., Cunningham, C. E., Boyle, M. H., Lingley-Pottie, P., Reid, G. J., Bagnell, A., Lipman, E. L., Turner, K., Corkum, P., Stewart, S. H., Berrigan, P., & Sdao-Jarvie, K. (2018). Distance-delivered parent training for childhood disruptive behavior (Strongest Families™): A randomized controlled trial and economic analysis. *Journal of Abnormal Child Psychology, 46*, 1613–1629. https://doi.org/10.1007/s10802-018-0413-y

Öztürk, Y., Özyurt, G., & Pekcanlar Aynur, A. (2019). The effects of the Triple P-Positive Parenting Programme on parenting, family functioning and symptoms of attention-deficit/hyperactivity disorder. A randomized controlled trial. *Psychiatry and Clinical Psychopharmacology, 29*(4), 665–673. https://doi.org/10.1080/24750573.2018.1542189

Paiva, G. C. de C., de Paula, J. J., Costa, D. de S., Alvim-Soares, A., Santos, D. A. F. e, Jales, J. S., Romano-Silva, M. A., & Miranda, D. M. de. (2024). Parent training for disruptive behavior symptoms in attention deficit hyperactivity disorder: A randomized clinical trial. *Frontiers in Psychology*, *15*. https://doi.org/10.3389/fpsyg.2024.1293244

Palmer, M., Beckley-Hoelscher, N., Shearer, J., Kostyrka-Allchorne, K., Robertson, O., Koch, M., Pearson, O., Slovak, P., Day, C., Byford, S., Goldsmith, K., Waite, P., Creswell, C., & Sonuga-Barke, E. J. S. (2023). The effectiveness and cost-effectiveness of a universal digital parenting intervention designed and implemented during the COVID-19 pandemic: Evidence from a rapid-implementation randomized controlled trial within a cohort. *Journal of Medical Internet Research*, *25*, e44079. https://doi.org/10.2196/44079

Parra-Cardona, J. R., Bybee, D., Sullivan, C. M., Rodríguez, M. M. D., Tams, L., & Bernal, G. (2017). Examining the impact of differential cultural adaptation with Latina/o immigrants exposed to adapted parent training interventions. *Journal of Consulting and Clinical psychology, 85*(1), 58–71. https://doi.org/10.1037/ccp0000160

Patterson, G. R., Chamberlain, P., & Reid, J. B. (1982). A comparative evaluation of a parent-training program. *Behavior Therapy, 13*(5), 638–650. https://doi.org/10.1016/S0005-7894(82)80021-X

Patterson, J., Barlow, J., Mockford, C., Klimes, I., Pyper, C., & Stewart-Brown, S. (2002). Improving mental health through parenting programmes: Block randomised controlled trial. *Archives of Disease in Childhood, 87*(6), 472–477. https://doi.org/10.1136/adc.87.6.472

Perrin, E. C., Sheldrick, R. C., McMenamy, J. M., Henson, B. S., & Carter, A. S. (2014). Improving parenting skills for families of young children in pediatric settings: A randomized clinical trial. *JAMA Pediatrics, 168*(1), 16–24. https://doi.org/10.1001/jamapediatrics.2013.2919

Pickering, J. A., Crane, M. E., Hong, J., Nickel, A., Chainey, C., & Sanders, M. R. (2023). A randomized controlled trial of a parenting program to improve sibling relationships. *Journal of Child and Family Studies, 32*(3), 1438–1451. https://doi.org/10.1007/s10826-023-02539-7

Pisterman, S., Firestone, P., McGrath, P., Goodman, J. T., Webster, I., Mallory, R., & Goffin, B. (1992). The role of parent training in treatment of preschoolers with ADDH. *American Journal of Orthopsychiatry, 62*(3), 397–408. https://doi.org/10.1037/h0079356

Pisterman, S., McGrath, P. J., Firestone, P., Goodman, J. T., & et al. (1989). Outcome of parent-mediated treatment of preschoolers with attention deficit disorder with hyperactivity. *Journal of Consulting and Clinical Psychology*, *57*(5), 628–635. https://doi.org/10.1037//0022-006x.57.5.628

Porzig-Drummond, R., Stevenson, R. J., & Stevenson, C. (2014). The 1-2-3 Magic parenting program and its effect on child problem behaviors and dysfunctional parenting: A randomized controlled trial. *Behaviour Research and Therapy, 58*, 52–64. https://doi.org/10.1016/j.brat.2014.05.004

Porzig-Drummond, R., Stevenson, R. J., & Stevenson, C. (2015). Preliminary evaluation of a self-directed video-based 1-2-3 Magic parenting program: A randomized controlled trial. *Behaviour Research and Therapy, 66*, 32–42. https://doi.org/10.1016/j.brat.2015.01.003

Reedtz, C., Handegård, B. H., & Mørch, W. T. (2011). Promoting positive parenting practices in primary pare: Outcomes and mechanisms of change in a randomized controlled risk reduction trial. *Scandinavian Journal of Psychology, 52*(2), 131–137. https://doi.org/10.1111/j.1467-9450.2010.00854.x

Rincón, P., Cova, F., Saldivia, S., Bustos, C., Grandón, P., Inostroza, C., Streiner, D., Bühring, V., & King, M. (2018). Effectiveness of a positive parental practices training program for Chilean preschoolers’ families: A randomized controlled trial. *Frontiers in Psychology, 9*, 1751. https://doi.org/10.3389/fpsyg.2018.01751

Roshan, M., Aghaseofi, A., Alipour, A., & Rezarei, A. (2017). Comparison of the effectiveness of two methods of treatment of parent-child interaction and coping mother therapy to reduce the severity of behavioral problems in children 3-6 years old. *Biannual Journal of Clinical Psychology & Personality, 14*(1), 111–123.

Sampaio, F., Sarkadi, A., Salari, R., Zethraeus, N., & Feldman, I. (2015). Cost and effects of a universal parenting programme delivered to parents of preschoolers. *The European Journal of Public Health, 25*(6), 1035–1042. https://doi.org/10.1093/eurpub/ckv106

Sanders, M. R., Baker, S., & Turner, K. M. (2012). A randomized controlled trial evaluating the efficacy of Triple P Online with parents of children with early-onset conduct problems. *Behaviour Research and Therapy, 50*(11), 675–684. https://doi.org/10.1016/j.brat.2012.07.004

Sanders, M. R., Markie-Dadds, C., Tully, L. A., & Bor, W. (2000a). The Triple P-Positive Parenting Program: A comparison of enhanced, standard, and self-directed behavioral family intervention for parents of children with early onset conduct problems. *Journal of Consulting and Clinical Psychology, 68*(4), 624–640. https://doi.org/10.1037/0022-006X.68.4.624

Sanders, M. R., Montgomery, D. T., & Brechman-Toussaint, M. L. (2000b). The mass media and the prevention of child behavior problems: The evaluation of a television series to promote positive outcomes for parents and their children. *The Journal of Child Psychology and Psychiatry and Allied Disciplines, 41*(7), 939–948. https://doi.org/10.1111/1469-7610.00681

Sanders, M. R., Stallman, H. M., & McHale, M. (2011). Workplace Triple P: A controlled evaluation of a parenting intervention for working parents. *Journal of Family Psychology, 25*(4), 581–590. https://doi.org/10.1037/a0024148

Santini, P. M., & Williams, L. C. (2017). A randomized controlled trial of an intervention program to Brazilian mothers who use corporal punishment. *Child Abuse & Neglect, 71*, 80–91. https://doi.org/10.1016/j.chiabu.2017.04.019

Sayger, T. V., Horne, A. M., Walker, J. M., & Passmore, J. L. (1988). Social learning family therapy with aggressive children: Treatment outcome and maintenance. *Journal of Family Psychology*, *1*(3), 261–285. https://doi.org/10.1037/h0080461

Scavenius, C., Chacko, A., Lindberg, M. R., Granski, M., Vardanian, M. M., Pontoppidan, M., Hansen, H., & Eiberg, M. (2020). Parent management training Oregon model and family-based services as usual for behavioral problems in youth: A national randomized controlled trial in Denmark. *Child Psychiatry & Human Development, 51*(5), 839–852. https://doi.org/10.1007/s10578-020-01028-y

Schappin, R., Wijnroks, L., Uniken Venema, M., Wijnberg-Williams, B., Veenstra, R., Koopman-Esseboom, C., Mulder-De Tollenaer, S., Van der Tweel, I., & Jongmans, M. (2013). Brief parenting intervention for parents of NICU graduates: A randomized, clinical trial of Primary Care Triple P. *BMC Pediatrics, 13*(1), 1–9. https://doi.org/10.1186/1471-2431-13-69

Schilling, S., French, B., Berkowitz, S. J., Dougherty, S. L., Scribano, P. V., & Wood, J. N. (2017). Child–Adult Relationship Enhancement in Primary Care (PriCARE): A randomized trial of a parent training for child behavior problems. *Academic Pediatrics, 17*(1), 53–60. https://doi.org/10.1016/j.acap.2016.06.009

Schuhmann, E. M., Foote, R. C., Eyberg, S. M., Boggs, S. R., & Algina, J. (1998). Efficacy of parent-child interaction therapy: Interim report of a randomized trial with short-term maintenance. *Journal of Clinical Child Psychology, 27*(1), 34–45. https://doi.org/10.1207/s15374424jccp2701_4

Scott, S., O’Connor, T. G., Futh, A., Matias, C., Price, J., & Doolan, M. (2010a). Impact of a parenting program in a high‐risk, multi‐ethnic community: The PALS trial. *Journal of Child Psychology and Psychiatry, 51*(12), 1331–1341. https://doi.org/10.1111/j.1469-7610.2010.02302.x

Scott, S., Sylva, K., Beckett, C., Doolan, M., Kallitsoglou, A., & Ford, T. (2014). *Which type of parenting programme best improves child behaviour and reading? Follow-up of the Helping Children Achieve trial*. Nuffield Foundation. https://www.nuffieldfoundation.org/wp-content/uploads/2019/12/HCA_follow_up_report_13_05_14.pdf

Scott, S., Sylva, K., Doolan, M., Price, J., Jacobs, B., Crook, C., & Landau, S. (2010b). Randomised controlled trial of parent groups for child antisocial behaviour targeting multiple risk factors: The SPOKES project. *Journal of Child Psychology and Psychiatry, 51*(1), 48–57. https://doi.org/10.1111/j.1469-7610.2009.02127.x

Shaw, D. S., Dishion, T. J., Supplee, L., Gardner, F., & Arnds, K. (2006). Randomized trial of a family-centered approach to the prevention of early conduct problems: 2-year effects of the family check-up in early childhood. *Journal of Consulting and Clinical Psychology, 74*(1), 1–9. https://doi.org/10.1037/0022-006X.74.1.1

Sheeber, L. B., & Johnson, J. H. (1994). Evaluation of a temperament-focused, parent-training program. *Journal of Clinical Child Psychology*, *23*(3), 249–259. https://doi.org/10.1207/s15374424jccp2303_3

Shimabukuro, S., Daley, D., Thompson, M., Laver-Bradbury, C., Lovern, K., & Tripp, G. (2020). Supporting Japanese mothers of children at risk for attention deficit hyperactivity disorder (ADHD): A small scale randomized control trial of well parent Japan. *Journal of Child and Family Studies, 29*, 1604–1616. https://doi.org/10.1007/s10826-020-01704-6

Sim, A., Puffer, E., Green, E., Chase, R., Zayzay, J., Garcia-Rolland, E., & Boone, L. (2014). *Parents make the difference: Findings from a randomized impact evaluation of a parenting program in rural Liberia*. International Rescue Committee. https://www.rescue.org/report/parents-make-difference-findings-randomized-impact-evaluation-parenting-program-rural

Skowron, E. A., Nekkanti, A. K., Skoranski, A. M., Scholtes, C. M., Lyons, E. R., Mills, K. L., Bard, D., Rock, A., Berkman, E., Bard, E., & Funderburk, B. W. (2024). Randomized trial of parent–child interaction therapy improves child-welfare parents’ behavior, self-regulation, and self-perceptions. *Journal of Consulting and Clinical Psychology*, *92*(2), 75–92. https://doi.org/10.1037/ccp0000859

Smallegange, E. S., Hermanns, J. M. A., & Oort, F. J. (2016). Evaluating the effectiveness of combining Home-Start and Triple P parenting support in the Netherlands. *Children and Youth Services Review, 68*, 178–186. https://doi.org/10.1016/j.childyouth.2016.07.009

Smith, G. C., Hayslip Jr, B., Hancock, G. R., Strieder, F. H., & Montoro-Rodriguez, J. (2018). A randomized clinical trial of interventions for improving well-being in custodial grandfamilies. *Journal of Family Psychology, 32*(6), 816–827 https://doi.org/10.1037/fam0000457

Solís-Cámara, P., Medina Cuevas, Y., & Díaz Romero, M. (2015). Análisis comparativo de predictores potenciales de prácticas disciplinarias severas con preescolares, antes y después de un entrenamiento para padres. *Acta Colombiana de Psicología, 18*(2), 139–150.

Solís-Cámara R, P., Salcido, P. C., Romero, M. D., & Aguirre, B. I. R. (2004). Efectos multidimensionales de un programa de crianza en la interacción recíproca entre padres y sus niños pequeńos con problemas de comportamiento. *Psicología Conductual Revista Internacional de Psicología Clínica de la Salud, 12*(2), 197–214.

Somech, L. Y., & Elizur, Y. (2012). Promoting self-regulation and cooperation in pre-kindergarten children with conduct problems: A randomized controlled trial. *Journal of the American Academy of Child & Adolescent Psychiatry, 51*(4), 412–422. https://doi.org/10.1016/j.jaac.2012.01.019

Sonuga-Barke, E. J., Barton, J., Daley, D., Hutchings, J., Maishman, T., Raftery, J., Stanton, L., Laver-Bradbury, C., Chorozoglou, M., Coghill, D., Little, L., Ruddock, M., Radford, M., Yao, G. L., Lee, L., Shipway, L. Markomichali, P., McGuirk, J., Lowe, M., . . . & Thompson, M. J. (2018). A comparison of the clinical effectiveness and cost of specialised individually delivered parent training for preschool attention-deficit/hyperactivity disorder and a generic, group-based programme: A multi-centre, randomised controlled trial of the New Forest Parenting Programme versus Incredible Years. *European Child & Adolescent Psychiatry, 27*, 797–809. https://doi.org/10.1007/s00787-017-1054-3

Sonuga-Barke, E. J., Daley, D., Thompson, M., Laver-Bradbury, C., & Weeks, A. (2001). Parent-based therapies for preschool attention-deficit/hyperactivity disorder: A randomized, controlled trial with a community sample. *Journal of the American Academy of Child & Adolescent Psychiatry, 40*(4), 402–408. https://doi.org/10.1097/00004583-200104000-00008

Sourander, A., McGrath, P. J., Ristkari, T., Cunningham, C., Huttunen, J., Lingley-Pottie, P., Hinkka-Yli-Salomäki, S., Kinnunen, M., Vuorio, J., Sinokki, A., Fossum, S., & Unruh, A. (2016). Internet-assisted parent training intervention for disruptive behavior in 4-year-old children: A randomized clinical trial. *JAMA Psychiatry, 73*(4), 378–387. https://doi.org/10.1001/jamapsychiatry.2015.3411

Spaccarelli, S., Cotler, S., & Penman, D. (1992). Problem-solving skills training as a supplement to behavioral parent training. *Cognitive Therapy and Research, 16*(2), 1–17. https://doi.org/10.1007/BF01172953

Spijkers, W., Jansen, D. E., & Reijneveld, S. A. (2013). Effectiveness of Primary Care Triple P on child psychosocial problems in preventive child healthcare: A randomized controlled trial. *BMC Medicine*, *11*(1). https://doi.org/10.1186/1741-7015-11-240

Stallman, H. M., & Sanders, M. R. (2014). A randomized controlled trial of Family Transitions Triple P: A group-administered parenting program to minimize the adverse effects of parental divorce on children. *Journal of Divorce & Remarriage, 55*(1), 33–48. https://doi.org/10.1080/10502556.2013.862091

Stattin, H., Enebrink, P., Özdemir, M., & Giannotta, F. (2015). A national evaluation of parenting programs in Sweden: The short-term effects using an RCT effectiveness design. *Journal of Consulting and Clinical Psychology, 83*(6), 1069–1084. https://doi.org/10.1037/a0039328

Stolk, M. N., Mesman, J., Van Zeijl, J., Alink, L. R., Bakermans-Kranenburg, M. J., Van IJzendoorn, M. H., Juffer, F., & Koot, H. M. (2008). Early parenting intervention: Family risk and first-time parenting related to intervention effectiveness. *Journal of Child and Family Studies, 17*(1), 55–83. https://doi.org/10.1007/s10826-007-9136-3

Strayhorn, J. M., & Weidman, C. S. (1989). Reduction of attention deficit and internalizing symptoms in preschoolers through parent-child interaction training. *Journal of the American Academy of Child & Adolescent Psychiatry*, *28*(6), 888–896. https://doi.org/10.1097/00004583-198911000-00013

Sumargi, A., Sofronoff, K., & Morawska, A. (2015). A randomized-controlled trial of the Triple P-Positive Parenting Program seminar series with Indonesian parents. *Child Psychiatry & Human Development, 46*(5), 749–761. https://doi.org/10.1007/s10578-014-0517-8

Sutton, C. (1992). Training parents to manage difficult children: A comparison of methods. *Behavioural and Cognitive Psychotherapy, 20*(2), 115–139. https://doi.org/10.1017/S0141347300016906

Swift, M. C., Roeger, L., Walmsley, C., Howard, S., Furber, G., & Allison, S. (2009). Rural children referred for conduct problems: Evaluation of a collaborative program. *Australian* *Journal of Primary Health, 15*(4), 335–340. https://doi.org/10.1071/PY09029

Sze-To, H. (unpub). *Developing a Hong Kong version of the "Happy Parenting Program - Round the clock" for parents who work long hours*. [Clinical Trial Registration]. ISRCTN. https://doi.org/10.1186/ISRCTN61113603

Taylor, T. K., Schmidt, F., Pepler, D., & Hodgins, C. (1998). A comparison of eclectic treatment with Webster-Stratton's parents and children series in a children's mental health center: A randomized controlled trial. *Behavior Therapy, 29*(2), 221–240. https://doi.org/10.1016/S0005-7894(98)80004-X

Thomas, R., & Zimmer‐Gembeck, M. J. (2011). Accumulating evidence for parent–child interaction therapy in the prevention of child maltreatment. *Child Development, 82*(1), 177–192. https://doi.org/10.1111/j.1467-8624.2010.01548.x

Thompson, M. J., Laver-Bradbury, C., Ayres, M., Le Poidevin, E., Mead, S., Dodds, C., Psychogiou, L., Bitsakou, P., Daley, D., Weeks, A., Miller Brotman, L., Abikoff, H., Thompson, P., & Sonuga-Barke, E. J. (2009). A small-scale randomized controlled trial of the revised new forest parenting programme for preschoolers with attention deficit hyperactivity disorder. *European Child & Adolescent Psychiatry, 18*(10), 605–616. https://doi.org/10.1007/s00787-009-0020-0

Tiwawatpakorn, N., Worachotekamjorn, J., & Tassanakijpanich, N. (2022). Effectiveness of parenting training on emotional and behavioral problems in first through fourth grade Thai children with ADHD: A randomized controlled study. *Psychological Reports*, *125*(5), 2470–2484. https://doi.org/10.1177/00332941211026846

Tully, L. A., & Hunt, C. (2017). A randomized controlled trial of a brief versus standard group parenting program for toddler aggression. *Aggressive Behavior, 43*(3), 291–303. https://doi.org/10.1002/ab.21689

Tuntipuchitanon, S., Kangwanthiti, I., Jirakran, K., Trairatvorakul, P., & Chonchaiya, W. (2024). Online positive parenting programme for promoting parenting competencies and skills: Randomised controlled trial. *Scientific Reports*, *14*(1). https://doi.org/10.1038/s41598-024-70842-4

Turner, K. M., Richards, M., & Sanders, M. R. (2007). Randomised clinical trial of a group parent education programme for Australian Indigenous families. *Journal of Paediatrics and Child Health, 43*(4), 243–251. https://doi.org/10.1111/j.1440-1754.2007.01053.x

Turner, K. M., & Sanders, M. R. (2006). Help when it’s needed first: A controlled evaluation of brief, preventive behavioral family intervention in a primary care setting. *Behavior Therapy, 37*(2), 131–142. https://doi.org/10.1016/j.beth.2005.05.004

Van Aar, J., Leijten, P., Overbeek, G., Thomaes, S., & Rothman, A. J. (2021). Does setting goals enhance parenting intervention effects? A field experiment. *Behavior Therapy, 52*(2), 418–429. https://doi.org/10.1016/j.beth.2020.05.004

Van den Hoofdakker, B. J., Van der Veen-Mulders, L., Sytema, S., Emmelkamp, P. M., Minderaa, R. B., & Nauta, M. H. (2007). Effectiveness of behavioral parent training for children with ADHD in routine clinical practice: A randomized controlled study. *Journal of the American Academy of Child & Adolescent Psychiatry, 46*(10), 1263–1271. https://doi.org/10.1097/chi.0b013e3181354bc2

Van Zeijl, J., Mesman, J., Van IJzendoorn, M. H., Bakermans-Kranenburg, M. J., Juffer, F., Stolk, M. N., Koot, H. M., & Alink, L. R. A. (2006). Attachment-based intervention for enhancing sensitive discipline in mothers of 1- to 3-year-old children at risk for externalizing behavior problems: A randomized controlled trial. *Journal of Consulting and Clinical Psychology*, *74*(6), 994–1005. https://doi.org/10.1037/0022-006x.74.6.994

Vardanian, M. M., Ramakrishnan, A., Peralta, S., Siddiqui, Y., Shah, S. P., Clark-Whitney, E., & Chacko, A. (2020). Clinically significant and reliable change: Comparing an evidence-based intervention to usual care. *Journal of Child and Family Studies, 29*, 921–933. https://doi.org/10.1007/s10826-019-01621-3

Wahler, R. G., & Meginnis, K. L. (1997). Strengthening child compliance through positive parenting practices: What works? *Journal of Clinical Child Psychology, 26*(4), 433–440. https://doi.org/10.1207/s15374424jccp2604_12

Walg, M. (2012). Wirksamkeit des" Kompetenztrainings für Eltern sozial auffälliger Kinder"(KES) aus Sicht von Erziehungspersonen und deren Kindern. [Doctoral dissertation, University of Cologne]

Ward, C. L., Wessels, I. M., Lachman, J. M., Hutchings, J., Cluver, L. D., Kassanjee, R., Nhapi, R., Little, F., & Gardner, F. (2020). Parenting for Lifelong Health for Young Children: A randomized controlled trial of a parenting program in South Africa to prevent harsh parenting and child conduct problems. *Journal of Child Psychology and Psychiatry, 61*(4), 503–512. https://doi.org/10.1111/jcpp.13129

Webster-Stratton, C. (1982). The long-term effects of a videotape modeling parent-training program: Comparison of immediate and 1-year follow-up results. *Behavior Therapy, 13*(5), 702–714. https://doi.org/10.1016/S0005-7894(82)80026-9

Webster-Stratton, C. (1984). Randomized trial of two parent-training programs for families with conduct-disordered children. *Journal of Consulting and Clinical Psychology, 52*(4), 666–678. https://doi.org/10.1037/0022-006X.52.4.666

Webster-Stratton, C. (1990). Enhancing the effectiveness of self-administered videotape parent training for families with conduct-problem children. *Journal of Abnormal Child Psychology, 18*(5), 479–492. https://doi.org/10.1007/BF00911103

Webster-Stratton, C. (1992). Individually administered videotape parent training: “Who benefits?” *Cognitive Therapy and Research, 16*(1), 31–52. https://doi.org/10.1007/BF01172955

Webster-Stratton, C., & Hammond, M. (1997). Treating children with early-onset conduct problems: A comparison of child and parent training interventions. *Journal of Consulting and Clinical Psychology, 65*(1), 93–109. https://doi.org/10.1037/0022-006X.65.1.93

Webster-Stratton, C., Kolpacoff, M., & Hollinsworth, T. (1988). Self-administered videotape therapy for families with conduct-problem children: Comparison with two cost-effective treatments and a control group. *Journal of Consulting and Clinical Psychology, 56*(4), 558–566. https://doi.org/10.1037/0022-006X.56.4.558

Webster-Stratton, C., Reid, M. J., & Hammond, M. (2004). Treating children with early-onset conduct problems: Intervention outcomes for parent, child, and teacher training. *Journal of Clinical Child and Adolescent Psychology, 33*(1), 105–124. https://doi.org/10.1207/S15374424JCCP3301_11

Weeland, J., Chhangur, R. R., Van der Giessen, D., Matthys, W., de Castro, B. O., & Overbeek, G. (2017). Intervention effectiveness of The Incredible Years: New insights into sociodemographic and intervention-based moderators. *Behavior Therapy, 48*(1), 1–18. https://doi.org/10.1016/j.beth.2016.08.002

Westwood, S., Faelling, J., & Sutton, C. (2021). ‘Catch Them Being Good’: Preliminary findings from a brief parenting intervention. *Early Child Development and Care, 191*(10), 1613–1623. https://doi.org/10.1080/03004430.2019.1663184

Wiggins, T. L., Sofronoff, K., & Sanders, M. R. (2009). Pathways Triple P‐Positive Parenting Program: Effects on parent‐child relationships and child behavior problems. *Family Process, 48*(4), 517–530. https://doi.org/10.1111/j.1545-5300.2009.01299.x

Williams, M. E., Hoare, Z., Owen, D. A., & Hutchings, J. (2020). Feasibility study of the enhancing parenting skills programme. *Journal of Child and Family Studies, 29*, 686–698. https://doi.org/10.1007/s10826-019-01581-8

Williamson, A. A., Knox, L., Guerra, N. G., & Williams, K. R. (2014). A pilot randomized trial of community-based parent training for immigrant Latina mothers. *American Journal of Community Psychology, 53*, 47–59. https://doi.org/10.1007/s10464-013-9612-4

Wolfe, D. A., Edwards, B., Manion, I., & Koverola, C. (1988). Early intervention for parents at risk of child abuse and neglect: A preliminary investigation. *Journal of Consulting and Clinical Psychology, 56*(1), 40–47. https://doi.org/10.1037/0022-006X.56.1.40

Wood, J. (2014). Parent program to improve child behavior problems. https://clinicaltrials.gov/ct2/show/NCT02049749

Wood, J. N., Kratchman, D., Scribano, P. V., Berkowitz, S., & Schilling, S. (2021). Improving child behaviors and parental stress: A randomized trial of child adult relationship enhancement in primary care. *Academic Pediatrics*, *21*(4), 629–637. https://doi.org/10.1016/j.acap.2020.08.002

Wu, D., Hung Lau, E. Y., & Power, T. G. (2024). Effectiveness of mixed-mode parenting training program during the COVID-19 pandemic: A quasi-experiment study in Hong Kong. *Children and Youth Services Review*, *156*, 107246. https://doi.org/10.1016/j.childyouth.2023.107246

Yao, A., Shimada, K., Kasaba, R., & Tomoda, A. (2022). Beneficial effects of behavioral parent training on inhibitory control in children with attention-deficit/hyperactivity disorder: A small-scale randomized controlled trial. *Frontiers in Psychiatry, 13*, 859249. https://doi.org/10.3389/fpsyt.2022.859249

Zangwill, W. M. (1983). An evaluation of a parent training program. *Child & Family Behavior Therapy*, *5*(4), 1–16. https://doi.org/10.1300/J019v05n04_01

Zargarinejad, G. (unpub). Comparing the effectiveness of group emotion-focused and behavioral interventions for mothers in children with Attention Deficit/Hyperactivity Disorder. https://en.irct.ir/trial/42199/pdf

Zhang, N., Zhang, J., Gewirtz, A. H., & Piehler, T. F. (2018). Improving parental emotion socialization in military families: Results of a randomized controlled trial. *Journal of Family Psychology, 32*(8), 1046–1056. https://doi.org/10.1037/fam000046
